# Efficacy of natural NF-κB inhibitors in the treatment of fibrosarcoma: an in vitro model study

**DOI:** 10.3389/fcell.2024.1476007

**Published:** 2024-12-23

**Authors:** Justyna Radzka, Agnieszka Gizak, Małgorzata Drąg-Zalesińska, Katarzyna Haczkiewicz-Leśniak, Michał Kulus, Anna Szewczyk, Wojciech Szlasa, Marzenna Podhorska-Okołów, Julita Kulbacka

**Affiliations:** ^1^ Department of Molecular Physiology and Neurobiology, Faculty of Biology, University of Wroclaw, Wroclaw, Poland; ^2^ Division of Histology and Embryology, Department of Human Morphology and Embryology, Faculty of Medicine, Wroclaw Medical University, Wroclaw, Poland; ^3^ Division of Ultrastructure Research, Department of Human Morphology and Embryology, Wroclaw Medical University, Wroclaw, Poland; ^4^ Department of Molecular and Cellular Biology, Faculty of Pharmacy, Wroclaw Medical University, Wroclaw, Poland; ^5^ Department of Immunology and Bioelectrochemistry, State Research Institute Centre for Innovative Medicine, Vilnius, Lithuania

**Keywords:** muscle cancer, berberine, cucurbitacin E (CurE), curcumin, biochanin A, caffeic acid phenethyl ester (CAPE)

## Abstract

**Introduction:**

NF-κB plays a pivotal role in the progression of cancers, including myosarcomas such as fibrosarcoma. Plants possess considerable potential for the provision of chemotherapeutic effects against cancer. The present study assessed, among others, the cytotoxicity, migration capacity and DNA damage induced by several natural compounds (berberine, curcumin, biochanin A, cucurbitacin E (CurE) and phenethyl caffeic acid (CAPE)) in cancer cells (WEHI-164) and normal muscle cells (L6).

**Methods:**

IC50 parameter was determined for all substances after 24-hour incubation. Molecular docking studies were performed to assess compound binding to cytoskeletal proteins. Neutral comet assay and immunocytochemical analysis were used to assess the intensity of apoptosis, and transmission electron microscopy was employed to validate these results at the ultrastructural level.

**Results and Discussion:**

The results showed that the tested compounds had a significantly increased cytotoxic effect on cancer cells compared to normal cells. Furthermore, molecular docking studies indicated that CAPE, biochanin A, and CurE could inhibit actin polymerization, suggesting their potential role in disrupting the cytoskeleton of cancer cells. Increased expression of caspase-3 and PARP-1 in WEHI-164 cells after treatment indicated the induction of apoptosis. Transmission electron microscopy confirmed the presence of cellular stress and vacuolation in cells treated with these compounds, with more pronounced effects observed in cancer cells compared to normal cells. The results indicate that natural NF-κB inhibitors may be capable of selectively targeting cancer cells, reducing their viability and inducing apoptosis while sparing normal cells. This selectivity is of great importance for the development of safer anticancer therapies. The results of this research support the hypothesis that these natural compounds may be effective anticancer agents, particularly in the treatment of fibrosarcoma. Further, in vivo studies and clinical trials are required to gain a full understanding of their mechanisms of action and potential synergies with existing chemotherapeutic agents.

## 1 Introduction

Cancer is currently a significant public health issue, ranking among the leading causes of death worldwide. Approximately 70% of cancer-related deaths occur in developing nations. According to estimates by the International Agency for Research on Cancer (IARC), the annual number of new cancer cases is projected to increase to around 21.7 million by 2030, with deaths reaching 13 million (Dalton et al., 2019). Among the various strategies for cancer treatment, chemotherapy is widely recognized as one of the most commonly used methods to prevent and treat cancer. However, current chemotherapeutic drugs have significant side effects and can lead to acquired drug resistance (Srivastava et al., 2016). Therefore, it is imperative to identify new, effective, and less toxic therapeutic agents for the treatment of cancer. Plants possess considerable potential for the provision of chemotherapeutic effects against cancer. Over 60% of anti-cancer drugs have been derived from natural sources, including plants, microorganisms, and marine organisms (Dey et al., 2019). The research presented in this work makes use of a number of substances derived from natural sources. Biochanin A (5,7-dihydroxy-4′-methoxyisoflavone) is an isoflavone that can be found, among others, in red clover (*Trifolium pratense* L.) (Yu et al., 2019). Biochanin A has been demonstrated to inhibit epithelial-mesenchymal transition and to reduce the proliferation rate of lung cancer cells. This is achieved by activating the Bcl-2 and caspase-3 pathways, in addition to regulating the expression of cell cycle-related proteins (Y. Li et al., 2018). The polyphenol compound curcumin, derived from the spice turmeric (*Curcuma longa*), has been demonstrated to inhibit the nuclear factor kappa-light-chain-enhancer of activated B cells (NF-κB) signalling pathway in a range of cancer types, including rhabdomyosarcoma. As a naturally occurring chemotherapeutic agent, curcumin has the potential to prevent and slow the progression of carcinogenesis. Additionally, it exhibits antioxidant and anti-inflammatory properties. The inhibition of factors responsible for cell proliferation and the inhibition of apoptosis, such as COX-2, AP-1, and NF-κB, is likely to occur as a result of the prevention of IκB phosphorylation by curcumin (Radzka et al., 2023). Berberine is a compound belonging to the isoquinoline alkaloid group that occurs naturally in a number of plant species, including those belonging to the Annonaceae*,* Berberidaceae, and Menispermaceae genera (Neag et al., 2018). Berberine exerts its effect by inhibiting the phosphorylation of the nuclear factor IκBα, which is responsible for activating NF-κB. Furthermore, evidence indicates that berberine treatment impairs the phosphorylation of the NF-κB subunit transcription factor p65. Berberine has been demonstrated to possess anti-cancer properties, whereby it downregulates caspase-1/IL-1β and also inhibits the cell cycle at the G1 phase in RMS cells (Jin et al., 2017). Caffeic acid phenethyl ester (CAPE) is a component identified in propolis. A substantial body of scientific literature attests to the antioxidant, anti-inflammatory, and cytotoxic effects of CAPE on cancer cells. It has been demonstrated that CAPE can inhibit NF-κB signaling by preventing the activation of upstream kinases, including IκB kinase (IKK) and TGF-β-activated kinase (TAK1), which are involved in the activation of the NF-κB pathway (Wang et al., 2010). Furthermore, evidence indicates that caffeic acid can inhibit the nuclear translocation of NF-κB and its binding to DNA, which are crucial steps in the transcriptional activation of NF-κB target genes (Abdel-Latif et al., 2005). Cucurbitacin E (CurE) is a triterpenoid compound that has been identified in a number of different plant species, including bitter melon (*Momordica charantia*) and cucumber (*Cucumis sativus*) (Chanda et al., 2020). This compound has been shown to modulate NF-κB signaling by inhibiting its activation and nuclear translocation (Qiao et al., 2013). Cucurbitacin E has been demonstrated to inhibit TNF-α-induced phosphorylation of inflammatory cytokines by modulating the NF-κB pathway. Additionally, cucurbitacin E inhibits the PI3K/Akt/mTOR pathway and the epithelial-mesenchymal transition (EMT) in osteosarcoma cells (Jia et al., 2015).

The NF-κB factor has been identified as the regulator of genes involved in tumour promotion, including those related to cell proliferation, angiogenesis, and adhesion. Furthermore, the activation of NF-κB has been demonstrated to influence the production of prostaglandins through the COX2 protein, which is overexpressed in a number of different types of cancer (Colotta et al., 2009). In neoplastic tissues, where elevated levels of the NF-κB factor are observed, there is an accumulation of pro-inflammatory cytokines, which contribute to the creation of a pro-neoplastic environment. Prolonged inflammation results in genomic instability and the emergence of genetic mutations that promote the formation and development of tumours (Elinav et al., 2013). Fibrosarcoma, an aggressive and poorly understood form of soft tissue sarcoma, poses significant therapeutic challenges, and recent evidence suggests that the NF-κB pathway may play a key role in its progression or angiogenesis (46). In this study, we demonstrated the effects of naturally occurring NF-κB inhibitors on fibrosarcoma cells, a cancer type where NF-κB’s role has not been extensively characterised in the context of these specific inhibitors. Although previous studies have investigated the activity of these compounds in various cancer models, their effects on fibrosarcoma remain underexplored, representing an important area of investigation. Fibrosarcoma is a particularly aggressive form of soft tissue sarcoma with a paucity of efficacious therapeutic options. Emerging evidence suggests that the NF-κB pathway plays a crucial role in its progression. By targeting NF-κB in fibrosarcoma, our objective is to provide new insights into potential therapeutic approaches for this malignancy. Apoptotic genes, including FLICE inhibitory protein, survivin, XIAP, c-IAP1/2 inhibitor, and the Bcl-2 family of proteins, are overexpressed in numerous types of cancer cells. The NF-κB factor induces this overexpression. It is therefore postulated that NF-κB plays a role in the regulation of anti-apoptotic mechanisms in the context of tumour formation (Kucharczak et al., 2003). A study of human prostate cancer cells (PC-3M cell line) has demonstrated that NF-κB plays a role in angiogenesis, invasion, and metastasis by regulating vascular endothelial growth factor (VEGF) and matrix metalloproteinases (MMP) (Huang et al., 2001). It has been demonstrated that the NF-κB factor exerts an influence on the resistance of cells to a range of chemotherapeutic agents. NF-κB plays a role in regulating the expression of the mdr1 gene, which is responsible for producing P-glycoprotein (P-gp). P-glycoprotein is a plasma membrane-associated multidrug transporter that affects the efflux of chemotherapy drugs, thereby contributing to treatment resistance (Wang et al., 2015). It has been demonstrated that NF-κB inhibits P-glycoprotein expression and may reactivate chemosensitivity. The involvement of the NF-κB factor in the degradation of muscle proteins has been demonstrated by research using cell cultures and in in vivo studies on laboratory animals (Wyke et al., 2004). In the study conducted by Moore–Carrasco et al., rats with hepatocellular carcinoma (AH-130 cell line) were treated with an NF-κB inhibitor (SP100030) and an activator protein-1 (AP-1) activator. The treatment was found to be effective in addressing skeletal muscle atrophy (Moore-Carrasco et al., 2007). Muscle wasting is a common occurrence in individuals of advanced age and those suffering from pathological conditions such as cancer. A study using a murine model indicated that the levels of NF-κB were elevated in the skeletal muscle tissues of animals with Lewis Lung Cancer (LLC). The study demonstrated that the transgenic overexpression of the super-repressor mutant protein IκBα (IκBαSR) resulted in a significant reduction in skeletal muscle and body weight loss in mice with LLC, which was achieved by inhibiting NF-κB activity (Cai et al., 2004). Recently, there has been a surge in interest in fructose 1,6-bisphosphatase (FBP) in the scientific community. This is a consequence of research which has demonstrated that FBP not only regulates the synthesis of glucose and glycogen from carbohydrate precursors, but also impacts (in a non-catalytic manner) a variety of cellular processes thanks to interactions with numerous proteins, e.g., mitochondrial VDAC, ANT, and ATP synthase, CAMK2, and transcription factors HIF1α and NF-κB. As a result of these interactions, FBP exerts influence over a number of cellular processes, including cell cycle-related events, mitochondrial biogenesis and membrane polarization, microtubule stability, glycolytic enzyme expression, synaptic plasticity, and even the progression of cancer (Gizak et al., 2012; Pirog et al., 2014). Mammalian tissues exhibit two distinct isoenzymes of fructose 1,6-bisphosphatase: liver FBP (FBP1) and muscle FBP (FBP2). The physiological function of FBP2 is modulated not only by its cellular expression levels but also by its oligomeric state. The dimeric form of FBP2 is associated with mitochondria, where it confers protection against cellular stress, while the tetrameric form is localized within the cell nucleus (Wiśniewski et al., 2017). FBP exerts negative effects on cell growth, and inhibits the progression of gastric cancer (Li et al., 2013). A study conducted in 2016 assessed the transcriptional levels of FBP1 and FBP2, revealing that elevated levels of FBP1 mRNA were associated with a favorable prognosis in gastric cancer. Subsequent verification of FBP1 protein expression confirmed that its overexpression correlated with improved clinical outcomes in patients with gastric cancer following surgical resection. Functional analyses further demonstrated that FBP1 expression significantly inhibited the proliferation and invasion of gastric cancer cells (Li et al., 2016). Additionally, FBP2 has been demonstrated to impede the progression of sarcomas by inhibiting mitochondrial biogenesis. It has been demonstrated that FBP2 impedes the progression of sarcoma by hindering mitochondrial biogenesis. Soft tissue sarcomas (STS) constitute a heterogeneous group of neoplasms that originate from connective tissues. Despite the considerable genetic heterogeneity observed among these tumours, an aberrant glucose metabolism is a common feature, although the underlying mechanism remains elusive. As was reported, FBP2 is absent in numerous STS subtypes, and reintroduction of FBP2 has been observed to significantly slow the growth of sarcomas. The researchers identified two distinct tumour-suppressing roles for FBP2 based on its location within the cell. Cytosolic FBP2 was observed to decrease glucose breakdown through its enzymatic function, while nuclear FBP2 was found to suppress a crucial factor involved in mitochondrial formation and function. The two roles of FBP2 result in a reduction in the energy supply to the cancer cells, which provides an explanation for the frequent loss of FBP2 in STS and its potential as a therapeutic target (Huangyang et al., 2020). In 2022, studies demonstrated that the chemically induced tetramerization of FBP2, resulting in a reduction of FBP2-mitochondria interactions, was associated with a range of changes in HL-1 cells. These included a decrease in mitochondrial membrane potential, disruption of the tubulin network and tubulin-mitochondria interactions, a marked reduction in mitochondrial membrane potential, and an increase in the speed and extent of mitophagy (Pietras et al., 2022). Furthermore, motor proteins, such as myosin, utilize ATP to generate force and facilitate movement along these actin filaments. This movement is essential for a number of cellular activities, including cell motility and structural integrity (Masters et al., 2017). The particular actions of myosins in relation to actin serve to illustrate their function in the organisation of the cytoskeleton and in the performance of cellular processes. Myosins, driven by the hydrolysis of ATP, interact with actin filaments, enabling their movement. This process is crucial for a range of cellular activities, including migration, division, and maintaining cellular shape (Nambiar et al., 2010). Recent research indicates that FBP2 is present in mitochondria under specific circumstances, where it interacts with a range of mitochondrial proteins. This mitochondrial localization is significant as it may affect processes such as fusion and fission, which are vital for maintaining the functionality and structural integrity of mitochondria. One investigation demonstrated that FBP2 interacts with proteins such as Voltage-Dependent Anion Channels (VDAC), Adenine Nucleotide Translocator (ANT), and elements of the mitochondrial ATP synthase complex. These interactions have the potential to influence mitochondrial membrane potential and regulate mitochondrial biogenesis. The presence of FBP2 in the mitochondria might also be involved in managing the mitochondrial response to stress, as well as processes such as mitochondrial trafficking and mitophagy (Collins, 2004). Furthermore, mitochondrial dynamics, which encompass the processes of fusion and fission, are vital for preserving mitochondrial structure, distribution, and functionality within cells. Fusion serves as a quality control mechanism, whereby the contents of partially damaged mitochondria are mixed. In contrast, fission is essential for the removal of damaged mitochondrial components and the distribution of mitochondria during cell division. The involvement of FBP2 in these processes indicates that it may have a broader role in cellular homeostasis and the response to metabolic changes (Reynolds, 1963). The results of our studies presented here support the hypothesis that natural compounds acting as NF-κB inhibitors may be effective anticancer agents, particularly in the treatment of fibrosarcoma.

## 2 Materials and methods

### 2.1 Cell cultures

Two cell lines were used in in vitro studies: WEHI-164 - fibrosarcoma cells isolated from mice and L6 – myogenic cell line isolated from skeletal muscles of rat (ATCC^®^, LGC Standards, Teddington, UK). The WEHI-164 cells were grown in RPMI culture medium supplemented with 10% fetal bovine serum (FBS, HyClone, Logan, UT, USA) and 1% penicillin/streptomycin (Sigma-Aldrich, Merck-Millipore, Poznań, Poland). The L6 cells were grown in DMEM culture medium (Dulbecco’s Modified Eagle’s Medium, Sigma-Aldrich, Merck-Millipore, Poznan, Poland) with 10% fetal bovine serum (FBS, HyClone, Logan, UT, USA), 1% penicillin/streptomycin (Sigma-Aldrich, Merck-Millipore, Poznan, Poland) and 1% L–glutamine (ThermoFisher, Alab, Poland). Cell culture of both types of cell lines was carried out at 37°C and 5% CO_2_. Both cell lines were negative for *mycoplasma* (MycoBlue *Mycoplasma* Detector, Vazyme Biotech, China). Cell passages were carried out 2–3 times a week when confluency was about 80%–90%. Cells were then removed from the flasks by trypsinization (trypsin 0.25% and EDTA 0.02%; IITD, Wroclaw, Poland) and washed with DPBS buffer (Sigma-Aldrich, Merck-Millipore, Poznan, Poland).

### 2.2 Preparation of drug solutions

CurE (number CAS 18444-66-1), biochanin A (number CAS 207-744-7), CAPE (number CAS 104594-70-9), curcumin (number CAS 458-37-7) and berberine (number CAS 633-65-8) were obtained from Sigma-Aldrich (Merck-Millipore, Poznan, Poland). All reagents were dissolved according to the manufacturer’s protocols. DMSO was used as a solvent. Then, immediately before the tests, the substances were diluted in DMEM/RPMI to obtain the concentrations necessary for assays. CurE was diluted to concentrations of 5; 3;2.5; 2;1; 0.5 µM. Biochanin A was diluted to concentrations of 30; 20; 10; 5;2; 0.5 µM. CAPE was diluted to concentrations of 50; 25; 15; 10; 5;1 µM. Curcumin and berberine were diluted to concentrations of 50; 25; 20; 15; 10; and 5 µM. DMEM/RPMI without drugs was used as a control.

### 2.3 MTT viability assay

Briefly, WEHI-164 and L6 cells were plated in 96-well microculture plates at 1x10^4^ cells/well and incubated with berberine, curcumin, biochanin A, CAPE, and CurE at the indicated concentrations and periods. The culture medium was then removed from each well and 100 µL/well of MTT reagent [3-(4,5-dimethylthiazol-2-yl)-2,5-diphenyltetrazolium bromide] (Sigma-Aldrich, USA) was added, and the cells were incubated for 2 h at 37°C. After incubation, acidified isopropanol (100 PL, 0.04 M HCl in 99.9% isopropanol) was added to dissolve the formed formazan crystals. The samples were completely dissolved using the pipette mixing technique. The absorbance value was measured at 570 nm using a GloMax^®^ Discover multimode microplate reader (Promega, Madison, WI, USA). The IC_50_ values were calculated using Quest Graph™ IC_50_ Calculator (AAT Bioquest, Inc, Sunnyvale, CA, USA). IC_50_ was determined with a non-linear model. The experiments were carried out in five replicates. The results are expressed as the percentage of viable cells relative to untreated control cells.

### 2.4 Molecular docking

Molecular simulation of CurE, biochanin A and CAPE docking to cytoskeletal proteins: tubulin (PDB: 1TUB) and actin (PDB: 1J6Z). Crystal structures of monomeric actin (PDB:1J6Z) and polymeric actin were obtained from the Protein Data Base (PDB) for the studies. Small molecule compounds were generated using Avogadro Software. Before simulations and screening, both structures were prepared using LigPrep and Protein Prepare. Analysis of the results was performed using Meastro and Canvas Software. The Glide Extra Precision (XP) docking was performed prior to the MM-GBSA calculations. All the data from the *in silico* experiments has been collected and analysed.

### 2.5 Neutral comet assay (NCA)

Detection of DNA fragmentation associated with apoptosis or the intermediate damage neutral comet assay method described by Collins was used (Collins, 2004). Briefly, after exposure to the examined compounds, WEHI-164/L6 cells (10^4^ cells/well) were subjected to a trypsinization process. In the next step, 1x10^5^/ml cells were washed with PBS chilled to 4°C, mixed with low-temperature melting agarose at a ratio of 1:10, and spread on a slide glass. Slides were submerged in precooled lysis buffer (2.5 M NaCl, 100 mM EDTA, pH 10, 10 mM Tris base, and 1% Triton X-100) at 4°C for 1 h. After lysis and rinsing, slides were equilibrated in TBE solution (40 mM Tris/boric acid, 2 mM EDTA, pH 8.3) and electrophoresed at 1.0 V/cm for 20 min and then silver staining was performed. For scoring the comet pattern, 100-200 nuclei were counted from each slide. The ranking of apoptotic comets was performed using the method developed by Collins (Collins, 2004).

### 2.6 Confocal microscopy studies

WEHI-164 and L6 cells were seeded on the microscopic cover slides, placed in a 6-well plate (Sarstedt, Germany) and incubated overnight for adhesion. The cells were then incubated for 24 h with one of the cytotoxic substances: biochanin A (5 µM), CAPE (25 µM), CurE (2.5 µM), curcumin (20 µM) and berberine (50 µM). Untreated cells were used as a control. Cells were fixed with 4% paraformaldehyde (PFA) in phosphate-buffered saline (PBS), permeabilized with 0.5% Triton X-100 in PBS (v/v) for 5 min, and blocked with 1% Bovine Serum Albumin (BSA) in PBS for 45 min at room temperature. All washing steps were performed using PBS. The following primary antibody was used: muscle FBPase (mouse, SC390209) antibody at a dilution of 1:250 in PBS. The following secondary antibodies were used: AlexaFluor488 (A11029) antibody at a dilution of 1:200 in PBS. Incubation with primary antibodies was conducted for 120 min and for 1 h with secondary antibodies at room temperature. For the imaging (Zeiss Axio Observer 7 SP stand for LSM 980) confocal laser scanning microscopes was used.

### 2.7 Immunocytochemical staining

WEHI-164 and L6 cells were seeded on 8-well slides (Thermo Scientific) and incubated overnight for adhesion. The cells were then incubated for 24 h with cytotoxic substances: biochanin A (5 µM), CAPE (25 µM), CurE (2.5 µM), curcumin (20 µM) or berberine (50 µM). Untreated cells were used as a control. The apoptotic activity was assessed by immunocytochemical determination of anti-caspase-3 (C8487) (Sigma-Aldrich, Merck-Millipore, Poznan, Poland) and PARP-1 (SC-74470) (Santa Cruz, USA Biotechnology), and antibodies were diluted in PBS (CAS-3 1:800; PARP-1 1:500). Then, the samples were fixed in 4% formalin, and antibodies were applied for 24 h at 4°C. Then, the slides were washed 3 × 5 min in PBS with Triton. Subsequently, the slides are incubated for 1 h with ImmPRESS Reagent Peroxidase Universal (Anti-mouse/rabbit Ig) (Vector Laboratories, Newark, USA). The slides were rinsed again in PBS for 5 min. Thereafter, the DAB + chromogen diluted in DAB + Substrate Buffer was added for 5 min (incubation in the darkness) and rinsed in distilled water for 10 min. Then, hematoxylin was used to counterstain nuclei (1 min). In the following steps, dehydration was performed by increasing the concentration of ethanol and xylene and mounted in DPX Mounting Medium (Fluka, Germany). The upright microscope (Olympus BX51, Shinjuku, Tokio, Japan) was used to examine the immunocytochemical reaction. Stained cell numbers were evaluated by counting 100 cells in 3 randomly selected fields. The results were judged to be positive if staining was observed in more than 5% of the cells. The intensity of immunohistochemical staining was evaluated as follows: (−) negative, (no reaction), (+)weak, (++) moderate, and (+++) strong.

### 2.8 Transmission electron microscope (TEM)

Ultrastructural analysis of L6 and WEHI-164 cell lines was performed. The cells were incubated for 24 h with cytotoxic substances: biochanin A (5 µM), CAPE (25 µM), CurE (2.5 µM), curcumin (20 µM) or berberine (50 µM). Untreated cells were used as a control. The cells were then fixed for 30 min in 2.5% (vol/vol) glutaraldehyde and 0.1M phosphate buffer (pH 7.4). After post-fixation in 1% (wt/vol) osmium tetroxide, cells were dehydrated through a graded series of acetone and embedded in Epon (Sigma Aldrich, St. Louis, MI, USA). The Epon blocks were cut on Reichert Ultracut E. Ultrathin sections were, contrasted with uranyl acetate and lead citrate according to the standard method (Kong et al., 2009) and examined with a Talos L120C (ThermoFisher).

### 2.9 Immunofluorescent studies

WEHI-164 and L6 cells were seeded on the cover microscopic slide, placed in a 6-well plate (Sarstedt, Germany), and incubated overnight for adhesion. The cells were then incubated for 24 h with cytotoxic substances: biochanin A (5 µM), CAPE (25 µM), CurE (2.5 µM), curcumin (20 µM) or berberine (50 µM). Untreated cells were used as a control. Cells were fixed with 4% paraformaldehyde (PFA) in phosphate-buffered saline (PBS) and permeabilized with 0.5% Triton X-100 in PBS (v/v) for 5 min. Following, the incubation with FBS was performed for 1 h at 37°C and 5% CO_2_. Next, the cells were washed with Triton-X100. The following primary antibodies were used: Anti-beta tubulin (rabbit, AB108342) antibody at a dilution 1:300 and Zyxin (mouse, MAB6977) antibody at a dilution of 1:300 in PBS. The following secondary antibodies were used: Anti-Mouse AlexFluor488 (AB150113) antibody at a dilution of 1:200 and AlexaFluor594 (A11012) antibody at a dilution of 1:200 in PBS. Primary antibody was added for 1-h incubation at 37°C and 5% CO_2_. Following, the cells were washed with PBS and the secondary antibody was added for 1-h incubation at room temperature in the dark. In the end, the samples were washed with PBS. For the nuclei visualization and cell mounting Fluoroshield™ (Sigma-Aldrich, St. Louis, MI, USA) with DAPI (4,6-diamidino-2-phenylindole) was applied. In the end, the samples were washed with PBS. The samples were observed on the Olympus IX53 microscope (40x, Olympus, Tokyo, Japan) after blue, red, and green laser excitations (depending on the fluorophore).

### 2.10 BCAA–Glo Assay

To measure changes in branched chain amino acids (leucine, isoleucine, valine), L6 and WEHI-164 cells were seeded in 96-well luminescent plates at 25,000 cells/well. Cells were then treated with berberine (50 µM), curcumin (20 µM), biochanin A (5 µM), CAPE (25 µM), or CurE (2.5 µM) for 24 h. The experiment was performed using BCAA-Glo Assay (JE9300, Promega, Madison, WI, USA). Following the completion of the compound treatment, the medium was removed and discarded, and the cells were washed three times with 200 μL of cold PBS. A volume of 25 μL of PBS was then added to the cells that had been washed. A negative control, comprising PBS without cells, was included to ascertain the background of the assay. A volume of 12.5 µL of 0.6N HCl was then added. The mixture was then combined by agitation of the plate for a period of 5 minutes. A volume of 12.5 µL of neutralisation buffer was then added. The mixture was then agitated for 1 minute. A volume of 50 µL of BCAA detection reagent, comprising reductase, NAD, leucine dehydrogenase, reductase substrate and luciferin detection solution, was then added. The solution was then incubated at room temperature for 1 hour. The luminescence value was measured using a GloMax^®^ Discover multimode microplate reader (Promega, Madison, WI, USA). The experiments were carried out in five replicates.

### 2.11 Glycogen levels detection by luminescent assay

To perform the glycogen sensing measurement, L6 and WEHI-164 cells were seeded in 96-well luminescent plates at 25,000 cells/well. Cells were then treated with berberine (50 µM), curcumin (20 µM), biochanin A (5 µM), CAPE (25 µM), or CurE (2.5 µM) for 24 h. The experiment was performed using Glycogen-Glo Assay (J5051, Promega, Madison, WI, USA). Following the completion of the compound treatment, the medium was removed and discarded, and the cells were washed three times with 200 μL of cold PBS. A volume of 30 µL of PBS was then added to the washed cells. A solution of 0.3 N HCl was then added to the cells in order to lyse them. The contents of the plate were mixed by agitation for a period of 5 minutes. A further 15 µL of Tris buffer (pH 8.0) was then added. The contents of the plate should be mixed by shaking for 1 minute. Twenty-5 μL of each sample, the positive control (glycogen standards in the same buffer as the samples) and the negative control (buffer only) were transferred to a well of a 96-well plate. A volume of 25 μL of the glucoamylase digestion solution, comprising glucoamylase and glucoamylase buffer, was added to each well. The samples were then incubated for 1 hour at room temperature. A volume of 50 µL of the glucose detection reagent, comprising reductase, NAD, reductase substrate, glucose dehydrogenase, and the luciferin detection solution, was added to each well. The plate was mixed by agitation for a period of 1 minute, after which it was incubated for 90 min at room temperature. The luminescence value was determined using a GloMax^®^ Discover multimode microplate reader (Promega, Madison, WI, USA). The experiments were conducted in five replicates. The signal-to-noise ratio (S/N) was calculated by dividing net luminescence (mean luminescence for the sample minus mean luminescence for the negative controls) by the standard deviation of the negative control.

### 2.12 Wound healing assay

Cells from the L6 and WEHI-164 lines were cultured in 96-well plates (8 × 10^4^) for 24 h until they formed a confluent layer. A scratch was made with a sterile 200 μL pipette tip across the cell monolayer. The medium was removed and the cells were washed twice with DPBS to remove contaminants from the separated cells. Then the DPBS was removed and 200 ul of substances were added to each well in the following concentrations: biochanin A (5 µM), CAPE (25 µM), CurE (2.5 µM), curcumin (20 µM) and berberine (50 µM). Untreated cells were used as a control. Photos were taken every 12 h to assess the crack closure until a monolayer of cells was obtained again. Cell cultures were observed and photographed under the CKX41 Olympus microscope (Tokyo, Japan). Software ImageJ (LOCI, University of Wisconsin) was used to quantify the areas of the closing gap.

### 2.13 Statistical analysis

The experiments were performed in 3 replicates. The statistical analysis was performed using the GraphPad Prism 8 (GraphPad Software Inc, San Diego, CA, USA). Data are expressed as mean ± SD (standard deviation) of the mean and were analysed by two-way ANOVA (analysis of variance), with *p* < 0.05 being considered statistically significant.

## 3 Results

### 3.1 Cytotoxicity of the examined substances and IC_50_ determination

**FIGURE 1:**
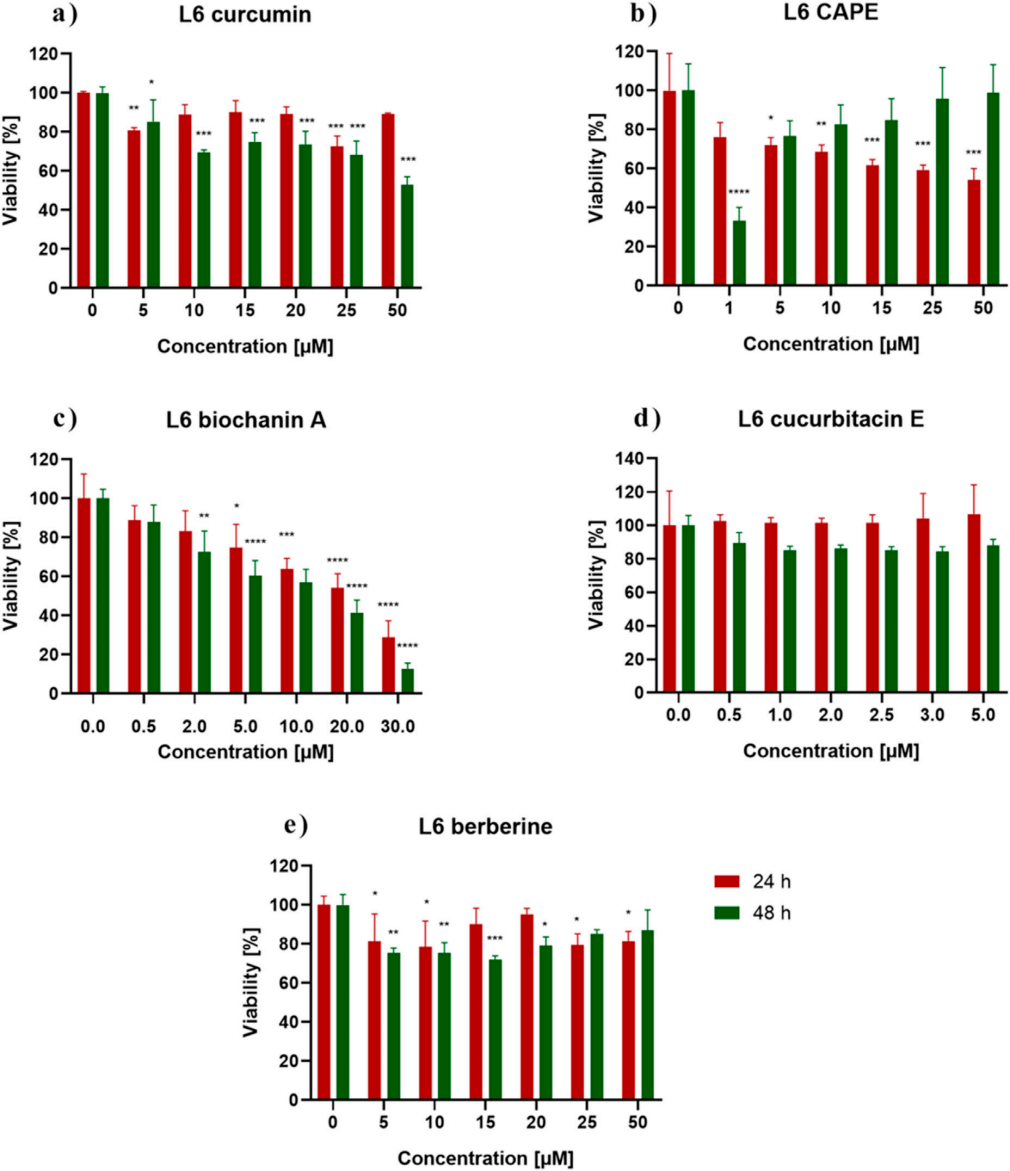
L6 cells viability measured by the MTT assay after 24 h and 48 h incubation with **(A)** berberine, **(B)** biochanin A, **(C)** CurE, **(D)** curcumin, **(E)** CAPE. Notes: (mean ± SD) N = 3, *p < 0.05, **p < 0.01, ***p < 0.005.

MTT assay assessed the cytotoxicity of the biochanin A, CAPE, CurE, curcumin and berberine. Figure 1 shows the response of L6 cells to 24 h and 48 h incubation with the analyzed substances. Incubation with berberine (e) and CurE (d) did not significantly change the mitochondrial activity of L6 cells compared to the untreated control group. In the case of incubation of L6 cells with biochanin A (c) it was observed that the cytotoxic effect increased with increasing concentration of the substance, both in the case of 24 h and 48 h incubation. At the highest concentration of 30 μM, cell survival after 24 h of incubation was ∼30% and after 48 h–18%. Incubation of cells with curcumin (a) showed an enhanced cytotoxic effect with prolonged incubation time (48 h). After exposure of cells to CAPE (b), the strongest cytotoxic effect was observed at a concentration of 1 µM (48 h). Then, with increasing CAPE concentration and prolonged incubation (48 h), cell survival increased, which can be possibly induced by the decay of CAPE in cells.

**FIGURE 2:**
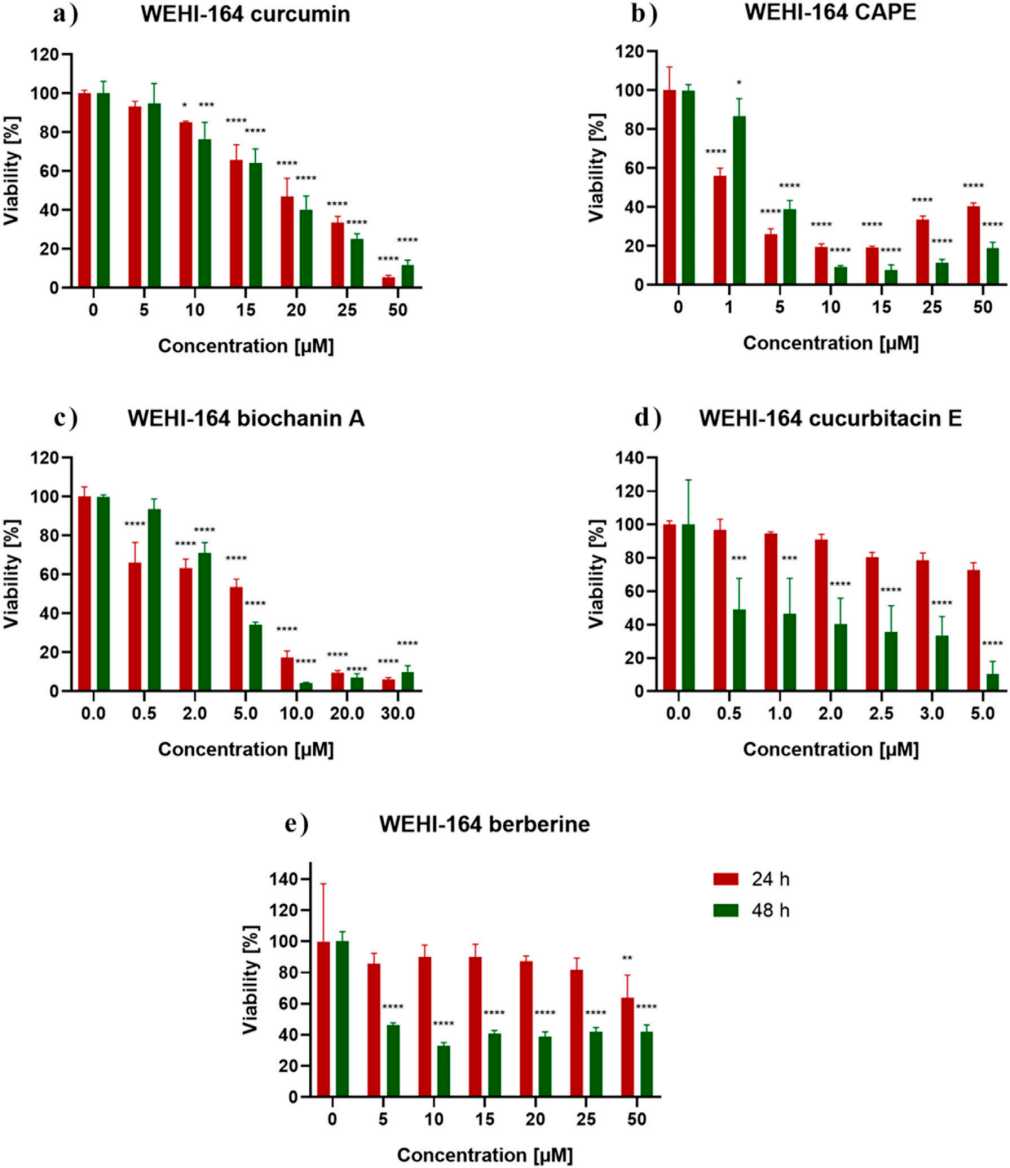
WEHI-164 cells viability measured by the MTT assay after 24 h and 48 h incubation with **(A)** berberine, **(B)** biochanin A, **(C)** CurE, **(D)** curcumin, **(E)** CAPE. Notes: (mean ± SD) N = 3, *p < 0.05, **p < 0.01, ***p < 0.005

Figure 2 shows the response of WEHI-164 cells to 24 h and 48 h incubation with the analyzed substances. Control represents the viability of untreated cells. WEHI-164 cells showed increased sensitivity to all of the tested substances. In the case of incubation with berberine (e), the strongest cytotoxic effect was obtained after 48 h of incubation at a concentration of 10 μM, where the percentage of cell survival was ∼33%. There was a clear decrease in the mitochondrial activity of cells incubated with biochanin A (c) at a concentration of 10 μM–30 µM and was ∼17%–9% (24 h). When incubated with CurE (d) and curcumin **(**a), cell survival decreased significantly with prolonged incubation and increasing concentration. All analysed substances had a stronger effect on cancer cells from the WEHI-164 line than on normal muscle cells from the L6 line. Table 1 presents the IC_50_ values calculated for biochanin A, curcumin, berberine, CAPE and CurE.

**TABLE 1:** IC50 values for the tested compounds determined after 24 h treatment of L6 and WEHI-164 cells.

Cell line/substance	Biochanin A	CAPE	CurE	Berberine	Curcumin
L6	5.903 [µM]	13.694 [µM]	10.38 [µM]	12.075 [µM]	29.305 [µM]
WEHI-164	6.395 [µM]	7.018 [µM]	66.476 [µM]	27.043 [µM]	14.049 [µM]

Based on the results presented above, low-toxic concentrations were selected for further experiment: biochanin A (5 µM), curcumin (20 µM), berberine (50 µM), CAPE (25 µM) and CurE (2.5 μM).

### 3.2 Apoptosis evaluation

**FIGURE 3:**
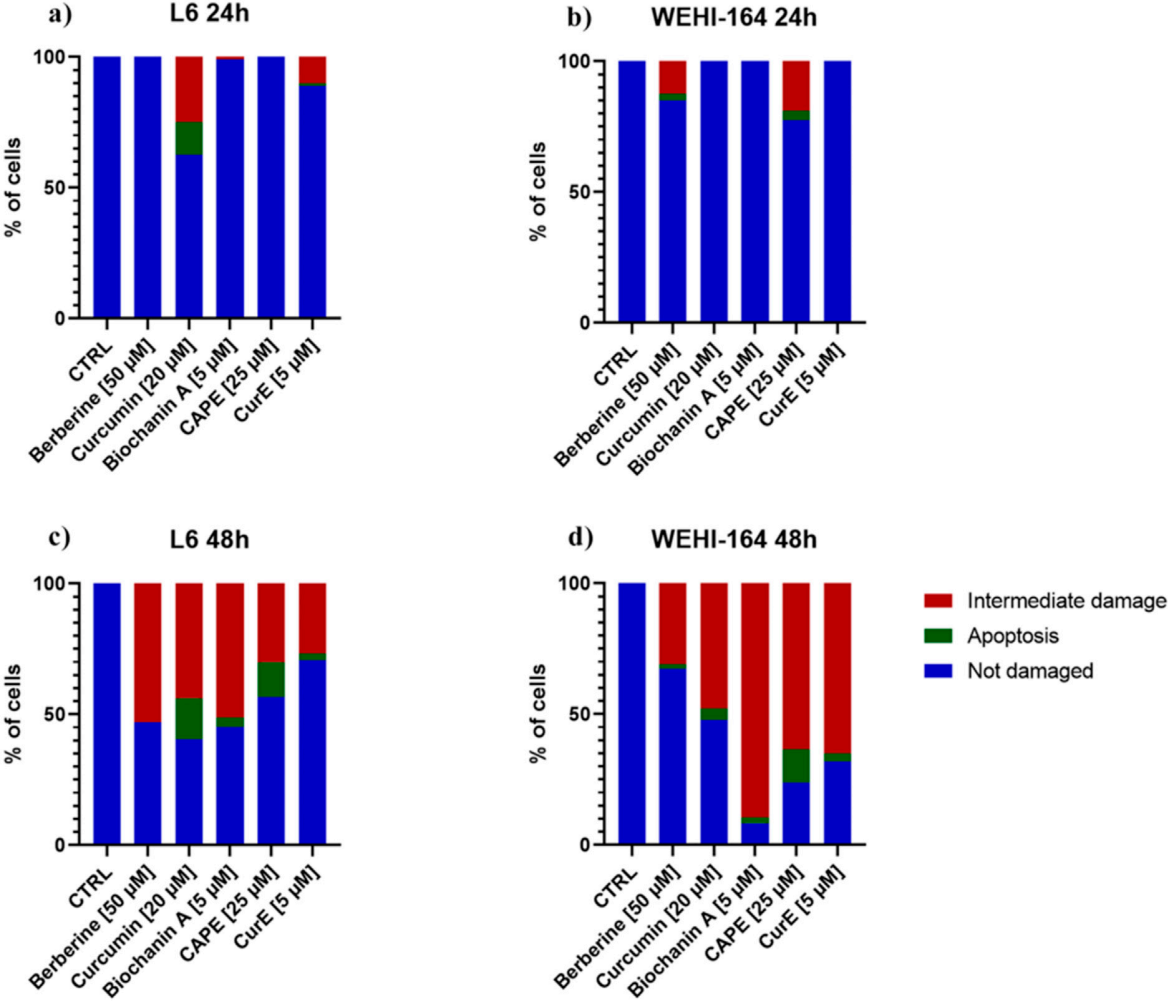
The neutral comet assay was performed on L6 and WEHI-164 cells after 24 h **(A, B)** and 48 h **(C, D)** of incubation with berberine, biochanin A, CurE, curcumin or CAPE.

**FIGURE 4:**
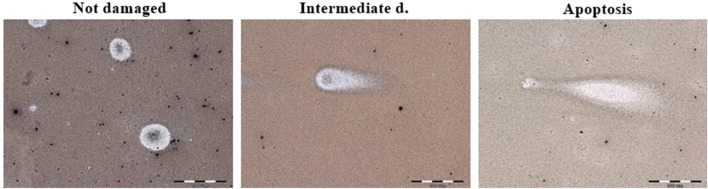
Example micrographs of assessed comets in not damaged, intermediately damaged and apoptotic states. Magnification ×600.

The apoptotic cell death was detected by neutral comet assay. The obtained results are demonstrated in Figures 3 and 4. Apoptotic cells in the L6 line were observed after 24 h of incubation with curcumin (12.5%) and CurE (1%) (a). No cell death was observed after exposure to the remaining substances. After 48 h of incubation of the L6 cells, apoptosis was observed in the case of incubation with curcumin (15.8%), CAPE (13.3%), biochanin A (3.6%) and CurE (2.5%) (c). In the case of WEHI-164 cells, apoptotic cells were observed after 24 h of incubation with CAPE (3.5%) and berberine (2.5%) (b). After 48 h of incubation of the WEHI-164 cells, apoptosis was observed after incubation with CAPE (12.7%), curcumin (4.4%), CurE (3.1%), biochanin A (2.2%) and berberine (1.6%) (d). There was a significant difference in the number of cells indirectly damaged after an extended (48 h) incubation time with the analysed substances. In the case of L6 cells, the highest percentage of damaged cells was observed following incubation with berberine (53%), while in the case of WEHI-164 cells, the highest percentage of damaged cells was observed following incubation with biochanin A (89.5%).

### 3.3 Immunocytochemical evaluation of PARP-1 and caspase-3

**FIGURE 5:**
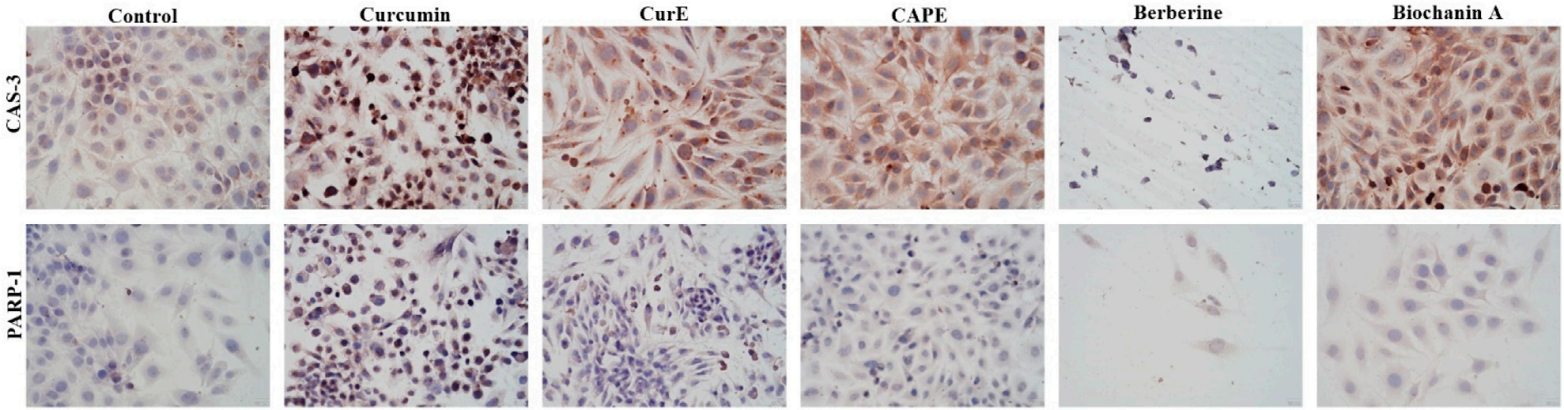
Caspase-3 and PARP-1 expression in L6 cells after 24 h incubation with curcumin, CurE, CAPE, berberine and biochanin A.

**FIGURE 6:**
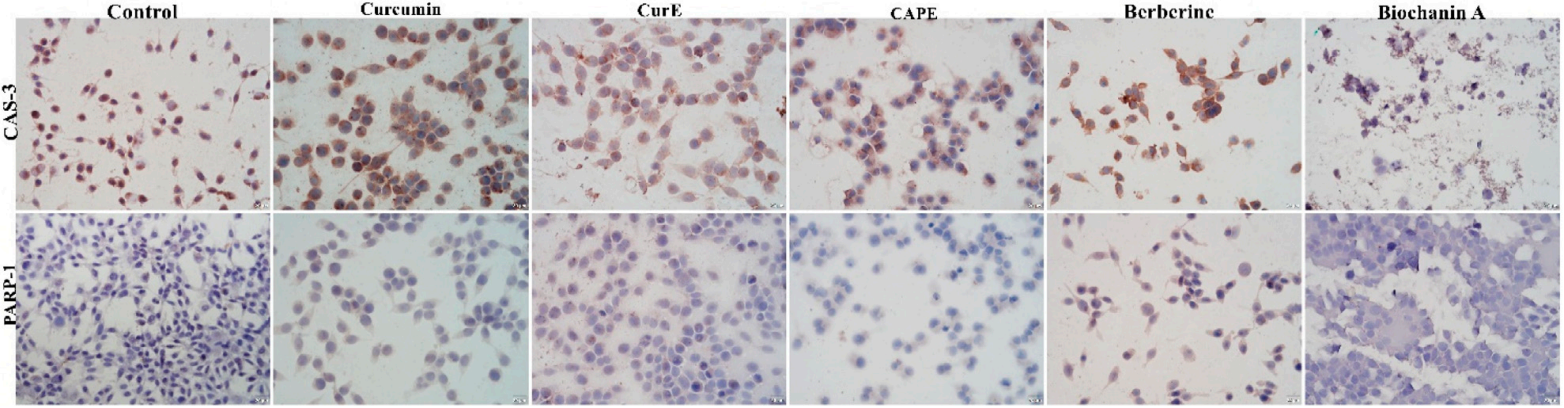
Caspase-3 and PARP-1 expression in WEHI-164 cells after 24 h incubation with curcumin, CurE, CAPE, berberine and biochanin A 1.3. Molecular Docking.

Immunocytochemical staining of PARP-1 and caspase-3 in L6 and WEHI-164 cells treated with biochanin A (5 µM), CAPE (25 µM), CurE (2.5 µM), curcumin (20 µM) or berberine (50 µM) was performed (Figures 5 and 6). PARP-1 is cleaved by caspase-3 during apoptosis, which leads to its inactivation (Wiśniewski et al., 2017), (Li et al., 2013). The results of immunocytochemical analyses of the apoptotic proteins are presented in Table 2. PARP-1 overexpression was observed in L6 cells after exposure to each compound in ca. 10%–15% of cells similarly to control cells. Caspase-3 expression was observed in ∼<5% of untreated L6 cells. In the case of incubation with biochanin A and berberine, the expression level of caspase-3 was increased in 45% of cells, and in other cases in 80%–90% of cells. PARP-1 overexpression was observed in WEHI-164 cells at a low level, i.e., in less than 5% of cells after berberine treatment and in untreated cells. After the exposure to biochanin A, CAPE and CurE, PARP-1 expression was increased in 40%–50% cells. The highest level of PARP-1 95% of cells, was noted after incubation with curcumin. Interestingly, in the case of control WEHI-164 cells caspase-3 expression was observed in 40% of cells, and after incubation with biochanin A, CAPE, CurE and curcumin it was detected in 85%–95% of cells. Only after the treatment with berberine, expression of caspase-3 was observed in less than 5% of cells.

**TABLE 2:** Evaluation of immunocytochemical reaction with anti-PARP-1 and anti-caspase 3 antibodies in L6 and WEHI-164 cells, after exposition to biochanin A (5 µM), CAPE (25 µM), CurE (2.5 µM), curcumin (20 µM) and berberine (50 µM) for 24 h

Compound	WEHI-164 cell line		L6 cell line	
	PARP-1	Caspase-3	PARP-1	Caspase-3
Control cells	<5%	40%, +	20%, +	<5%
Biochanin A [5 µM]	40%, +	95%, +++	15%, +	45%, +
CAPE [25 µM]	50%, +	95%, +++	10%, +	80%, ++
CurE [2.5 µM]	50%, +	85%, ++	15%, +	90%, ++
Curcumin [20 µM]	95%, +++	95%, +++	20%, +	80%, ++
Berberine [50 µM]	<5%	<5%	15%, +	45%, ++

### 3.4 Molecular Docking

**FIGURE 7:**
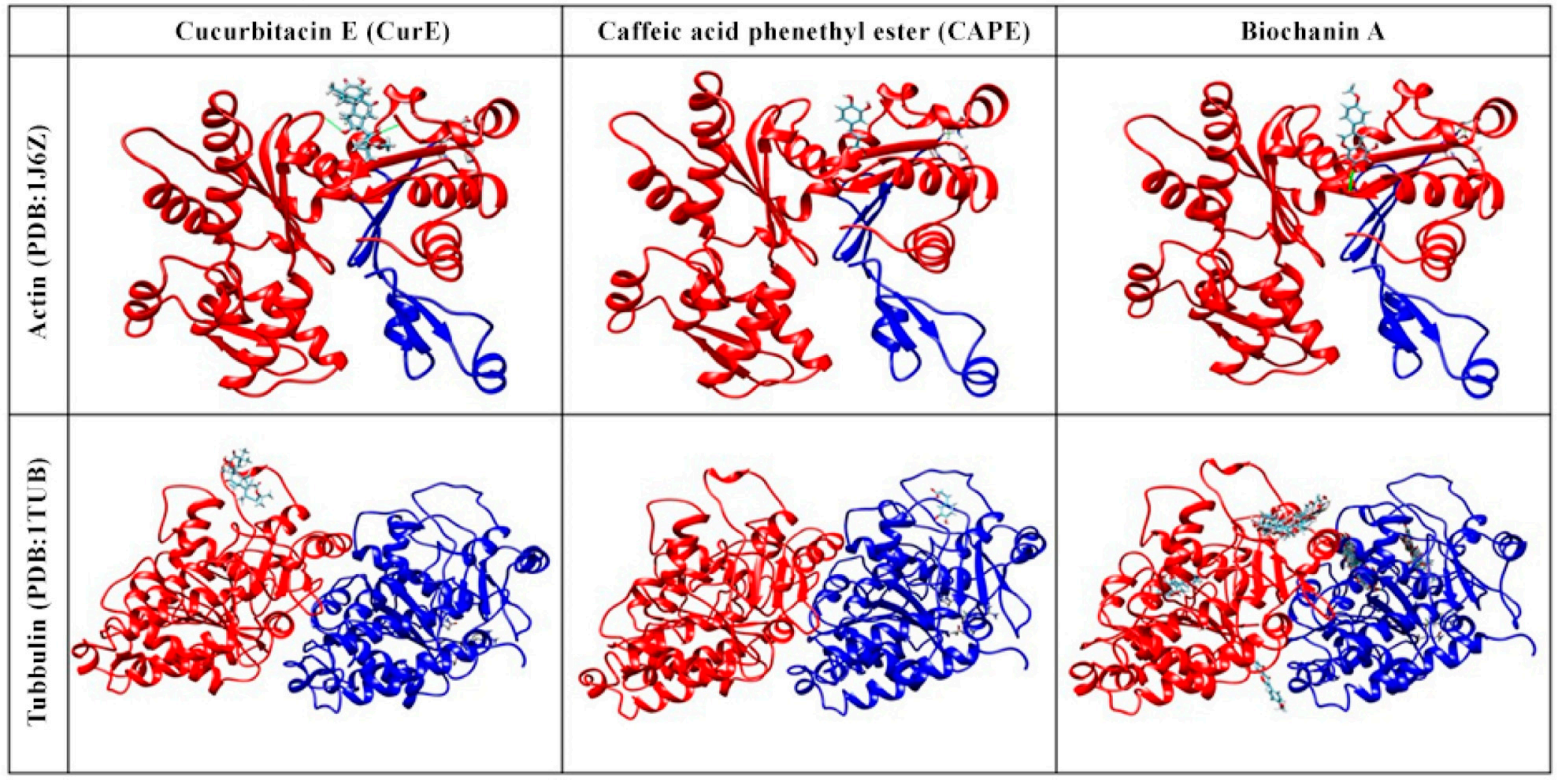
Molecular docking of actin (PDB:1J6Z) and tubulin (PDB:1TUB) with CAPE, biochanin A and CurE.

The obtained results indicate that CAPE, biochanin A and CurE showed anticancer activity (Figure 7). The compounds are also NF-κB and cell cycle inhibitors and therefore should be considered in the treatment of muscle cancers. According to molecular docking, the most likely binding site corresponds to the Taxotere (TMR) binding site (Dey et al., 2019). TMR is an agent that inhibitors actin polymerization, thus the same site of CAPE and biochanin A binding may indicate that the substances will extend similar effect on actin and tubulin.

Berberine does not act specifically on actin or tubulin but interacts with intracellular kinases (AMP-activated kinases). This is an important distinction because the results of molecular docking of berberine to actin and tubulin would not reflect the actual mechanism of action of this compound (Alakurtti et al., 2006).

Curcumin, the active component of turmeric, is known for its potential anti-cancer, anti-inflammatory, and antioxidant properties. However, its interactions with tubulin and actin, key cytoskeletal proteins, have not been extensively studied. This is due to the high binding specificity required by these proteins, which may not be achieved by curcumin’s broad spectrum of activity. Additionally, the dynamic nature of the cytoskeleton makes experimental confirmation of these interactions challenging. Focusing solely on docking with tubulin and actin may not fully reflect curcumin’s anti-cancer mechanism, considering its interactions with multiple signalling pathways and molecular targets. These factors limit the attractiveness of curcumin as a targeted therapeutic agent for cytoskeletal protein interactions.

In addition to NF-κB, we included tubulin as a secondary target in our molecular docking studies. Tubulin was selected due to its well-established role in cancer cell division and its relevance as a therapeutic target. Inhibiting tubulin can disrupt the mitotic process, which may enhance the anti-proliferative effects of NF-κB inhibition. Although this dual-target approach provides a promising strategy for cancer treatment, it should be noted that molecular docking, while useful for predicting potential binding interactions, offers only preliminary insights.

### 3.5 Alternations in cell ultrastructure–TEM study

**FIGURE 8:**
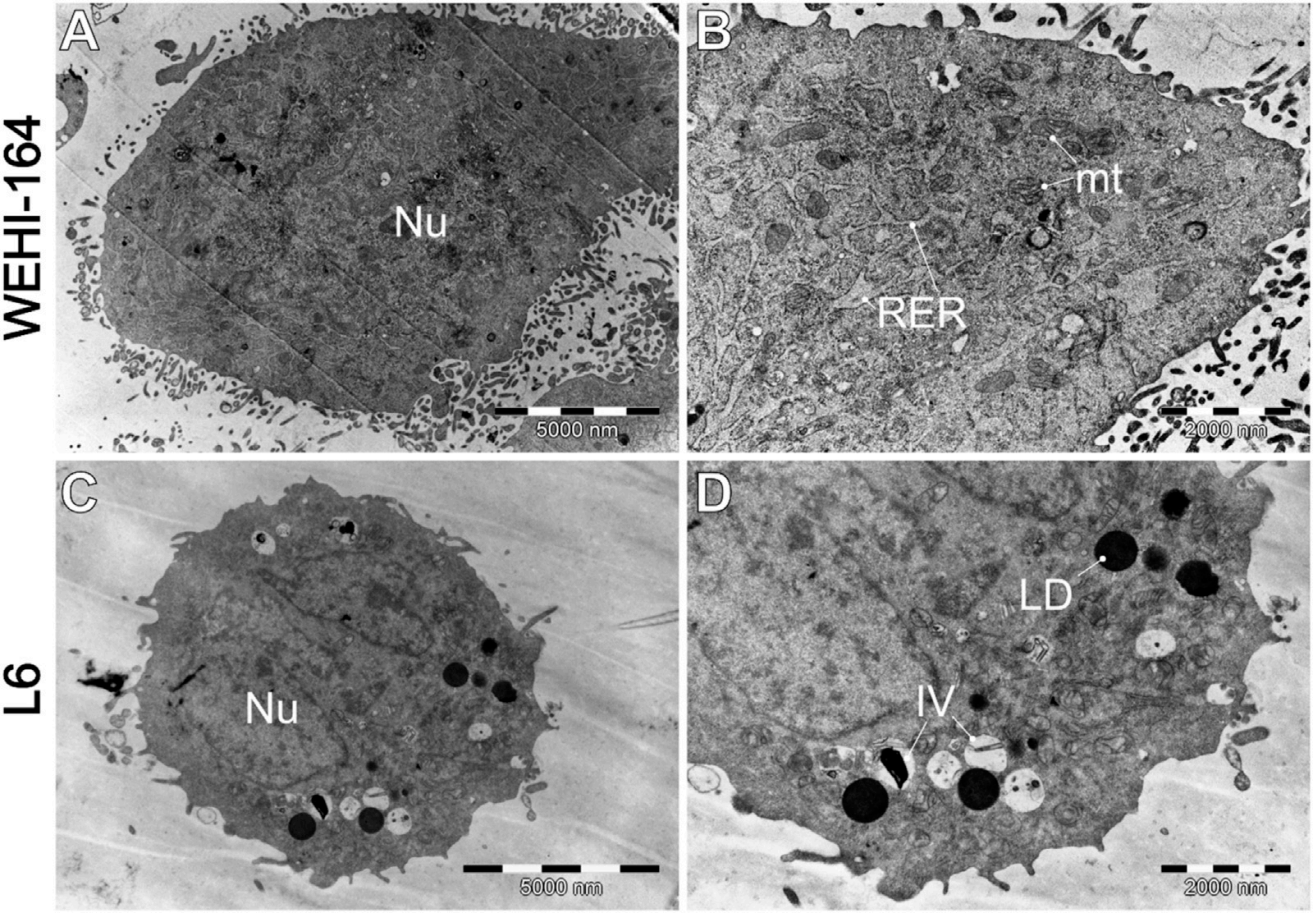
Morphology of untreated cells, where **(A, B)** WEHI-164, and **(C, D)** L6 cells. IV–intracellular vesicles; LD–lipid droplets; mt–mitochondria; Nu–cell nucleus; RER–rough endoplasmic reticulum.

**FIGURE 9:**
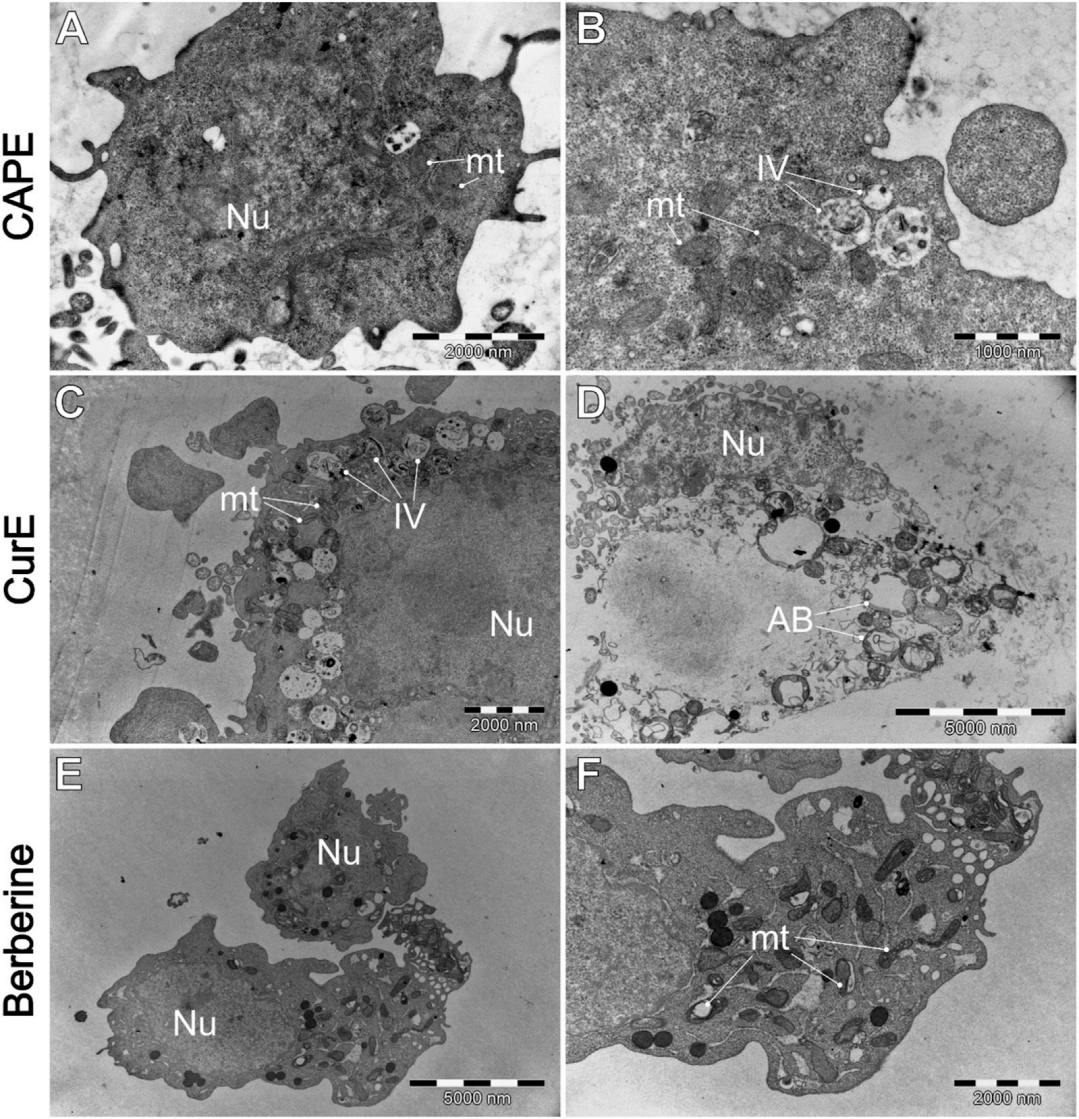
WEHI-164 cells’ morphology after the exposure to **(A, B)** CAPE (25 µM), **(C, D)** CurE (5 µM), **(E, F)** berberine (50 µM). AB–apoptotic bodies; IV–intracellular vesicles; mt–mitochondria; Nu–cell nucleus.

**FIGURE 10:**
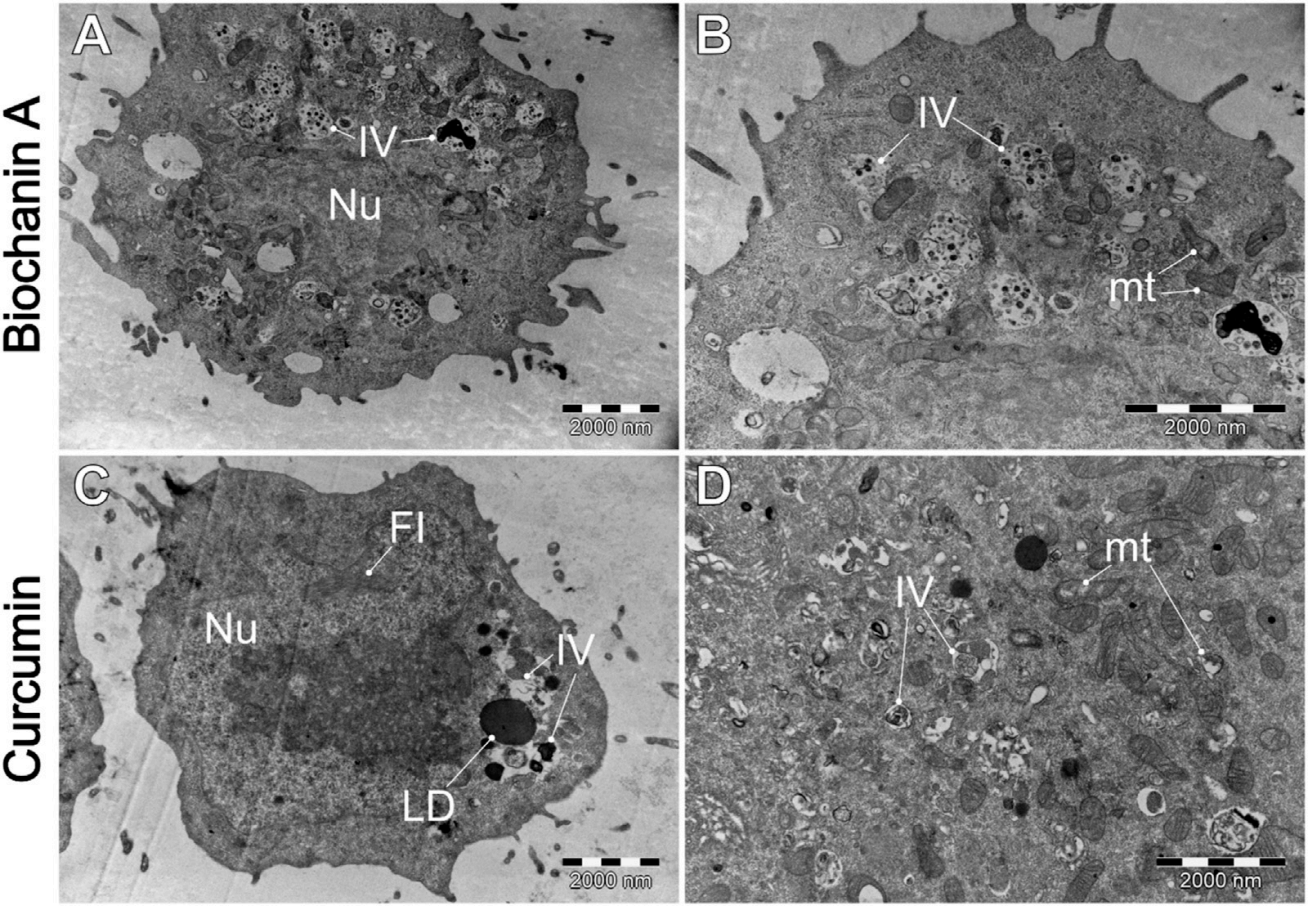
A comparative analysis of the morphological characteristics of WEHI-164 cells treated with **(A, B)** biochanin A (5 µM) and **(C, D)** curcumin (20 µM). IV–intracellular vesicles; LD–lipid droplets; mt–mitochondria; Nu–cell nucleus.

**FIGURE 11:**
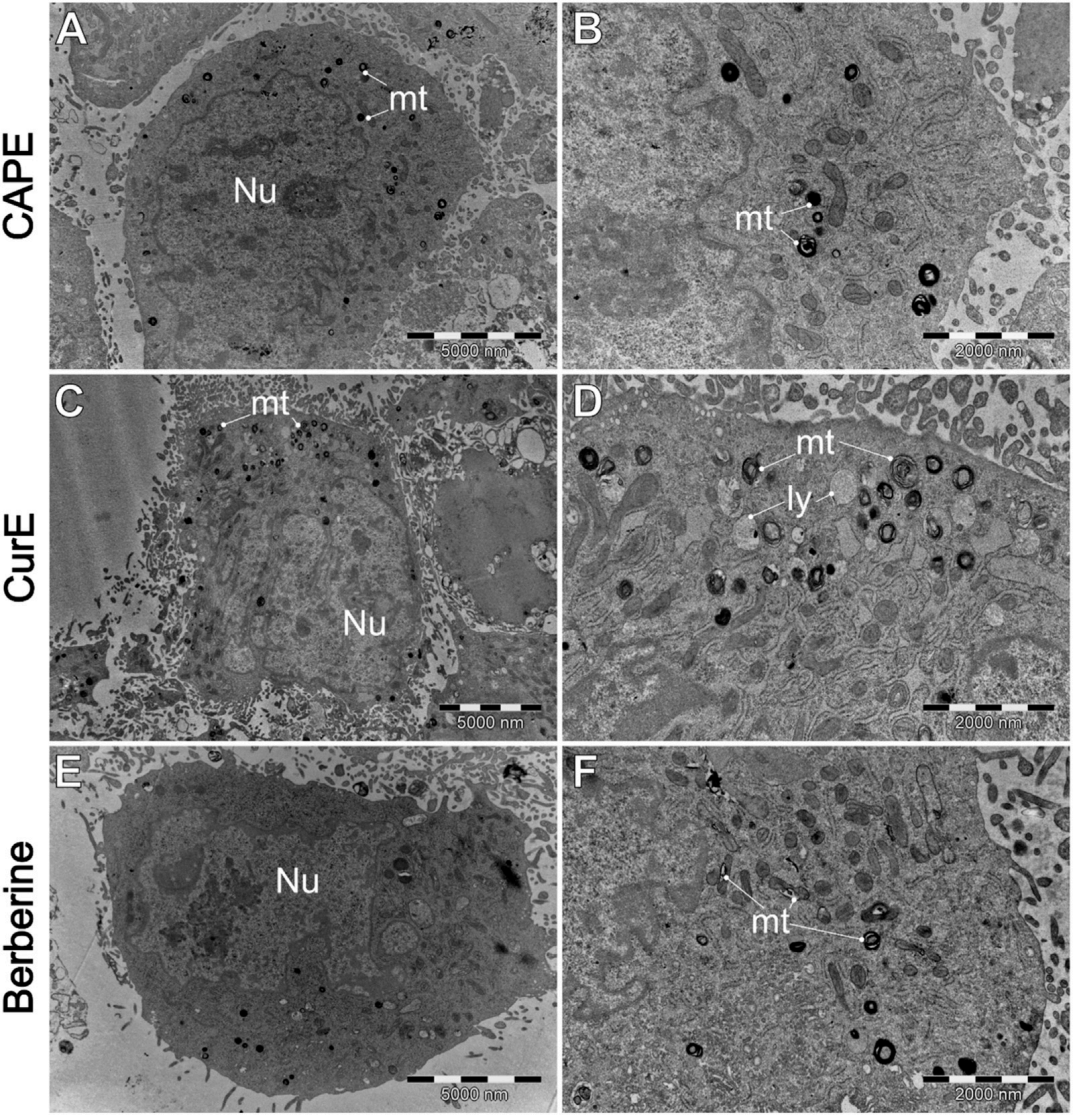
A comparison of the ultrastructure o L6 cells, treated by **(A, B)** CAPE (25 µM), **(C, D)** CurE (5 µM) and berberine **(E, F)** (50 µM). IV–intracellular vesicles; LD–lipid droplets; ly–lysosomes. mt–mitochondria; Nu–cell nucleus.

**FIGURE 12:**
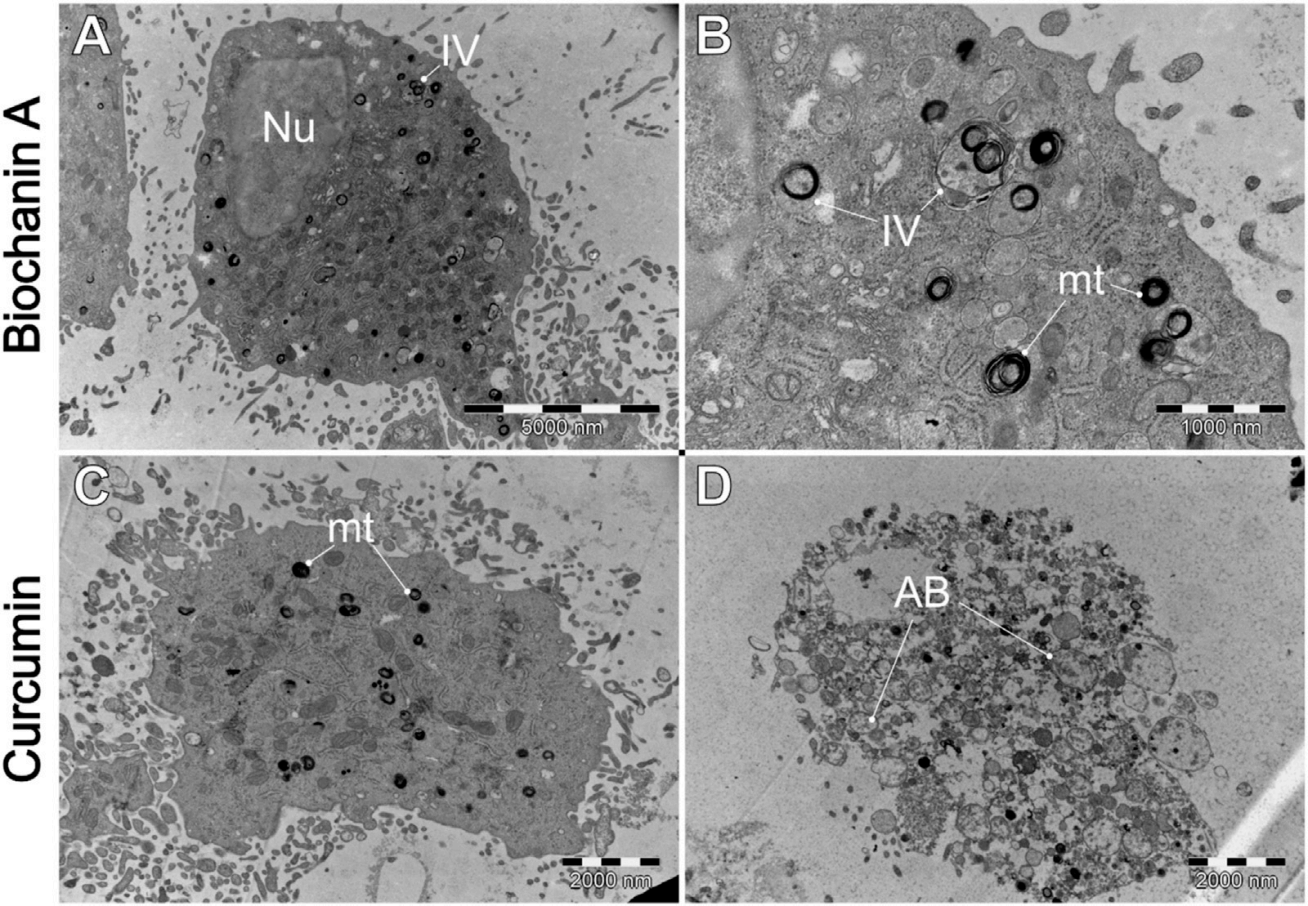
Ultrastructural effects of **(A, B)** biochanin A (5 µM) and **(C, D)** curcumin (20 µM) on L6 cells. AB–apoptotic bodies. IV–intracellular vesicles. mt–mitochondria. Nu–cell nucleus.

Transmission electron microscopy (TEM) was employed to investigate the ultrastructural effects of biochanin A, curcumin, and berberine on sarcoma (WEHI-164) and normal muscle (L6) cells. This analysis aimed to elucidate the cellular stress mechanisms and morphological changes induced by these natural compounds, providing insights into their potential anticancer activity. By comparing treated and untreated cells, we were able to discern distinct cytotoxic and protective effects, highlighting the differential responses of normal and cancerous cells to these agents. The results are shown in Figures 8–12 below.

Figure 8 shows that cells typically exhibit a particularly dark cytoplasm, a regular cell membrane, a barely discernible cell nucleus (Nu), a well-developed rough endoplasmic reticulum (RER), and undamaged mitochondria (mt). It is noteworthy that WEHI-164 cells typically exhibit a minimal number of intracellular vesicles. In comparison, L6 (C and D) cells display a less electron-dense cytoplasm with a predominantly euchromatic nucleus. Additionally, they often contain numerous lipid droplets (LD) and intracellular vesicles (IV), including phagolysosomes and autophagolysosomes with visible lamellar cellular debris.

Figure 9 demonstrates the impact of natural drug exposure on cancer cell morphology. CAPE did not significantly affected cell structure. The general morphology of the cells remains unaltered, and the mitochondria are mostly unaffected. However, there is an slightly increase in the number of intracellular vesicles (IV). Administration of CurE results (Figures 9C, D) in more pronounced alterations. The images demonstrate the presence of numerous intracellular vesicles, a blebbing plasma membrane with intact organelles inside blebs. The presented images suggest the occurrence of early (C) and late (D) apoptotic events, as evidenced by the presence of damaged apoptotic bodies (AB). The exposure of WEHI-164 cells to berberine (Figures 9E, F) resulted in an increased number of visibly damaged mitochondria (mt) and visible distortion of the cell membrane.

As we can observe biochanin A induced an increased number of intracellular vesicles (IV), yet no other significant alterations in mitochondria or cell membrane are observed. Cells cultured with curcumin exhibited a slightly elevated number of intracellular vesicles and, on occasion, lipid droplets. Some mitochondrial damage is evident, though not pervasive.

Figure 11 shows the L6 cells ultrastructure after the exposure to natural drugs. As we can see CAPE induced visible mitochondrial damage, with the presence of numerous lamellized mitochondrial debris. However, there is no evidence of mitophagy or autophagy when cells were treated by CurE. Berberine treatment induced similar alternations.

The exposure of L6 to biochanin A induced comparable events to those observed in Figure 11, with addition to spontaneous mitochondrial damage, and the presence of autophagosomes were observed. These findings suggest that biochanin A induces cellular stress leading to autophagic activity. Curcumin appears to induce the most pronounced changes, including distortion of the cell membrane (C) and an increased number of cellular debris (D), including evidence of programmed cell death.

We suspect these cellular structures appear to be autophagosomes. Autophagosomes are double-membrane vesicles involved in the degradation and recycling of cellular components through the process of autophagy. These structures are typically seen as spherical or cup-shaped with distinctive membranes, and appear when cells are exposed to stress, e.g., in response to therapy (Yun and Lee, 2018).

Our results indicate that exposure of WEHI-164 cells to berberine (Figures 9E, F) highlighted severe cellular damage, suggesting apoptotic or autophagic processes. The results obtained from TEM analysis revealed differential responses between normal muscle L6 cells and sarcoma WEHI-164 cells to natural anticancer drugs. Biochanin A and berberine induced significant vacuolization and cellular stress in both cell types, with more pronounced effects in cancerous cells. Curcumin appeared to have a more selective impact, causing notable morphological changes in WEHI-164 cells while preserving L6 cell integrity. These findings underscore the potential of these natural compounds in cancer therapy, with varying degrees of cytotoxicity and cellular responses observed under electron microscopy.

### 3.6 Immunofluorescent visualization of tubulin and zyxin reorganization and FBPase distribution

**FIGURE 13:**
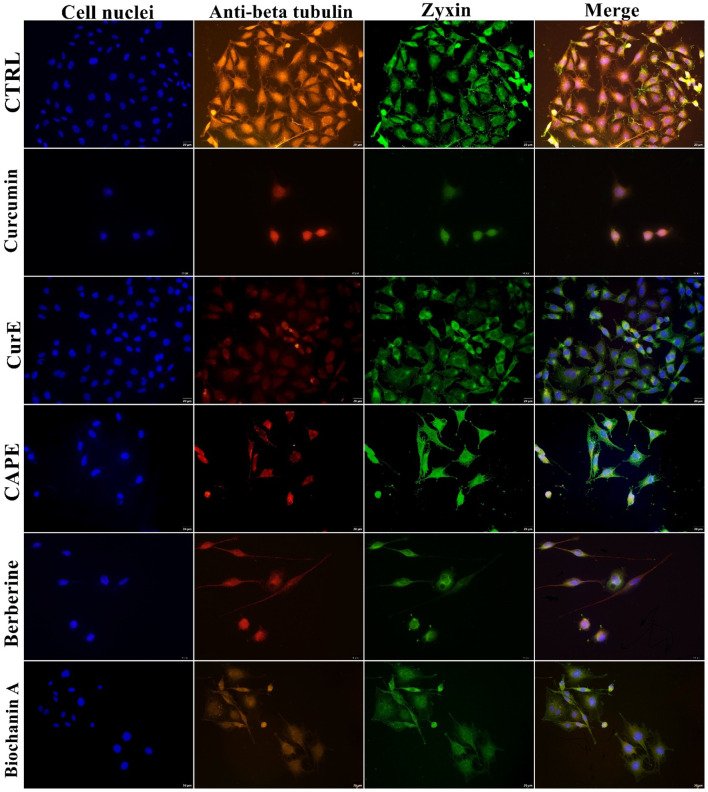
Presentation of the effect of curcumin (20 µM), CurE (2.5 µM), CAPE (25 µM), berberine (50 µM) and biochanin A (5 µM) on L6 cells after 24 h of incubation. Nuclei are stained blue with DAPI, zyxin is stained green with AlexaFluor488 and anti-beta tubulin antibody is stained red with AlexaFluor594. Scale bar = 20 μm.

**FIGURE 14:**
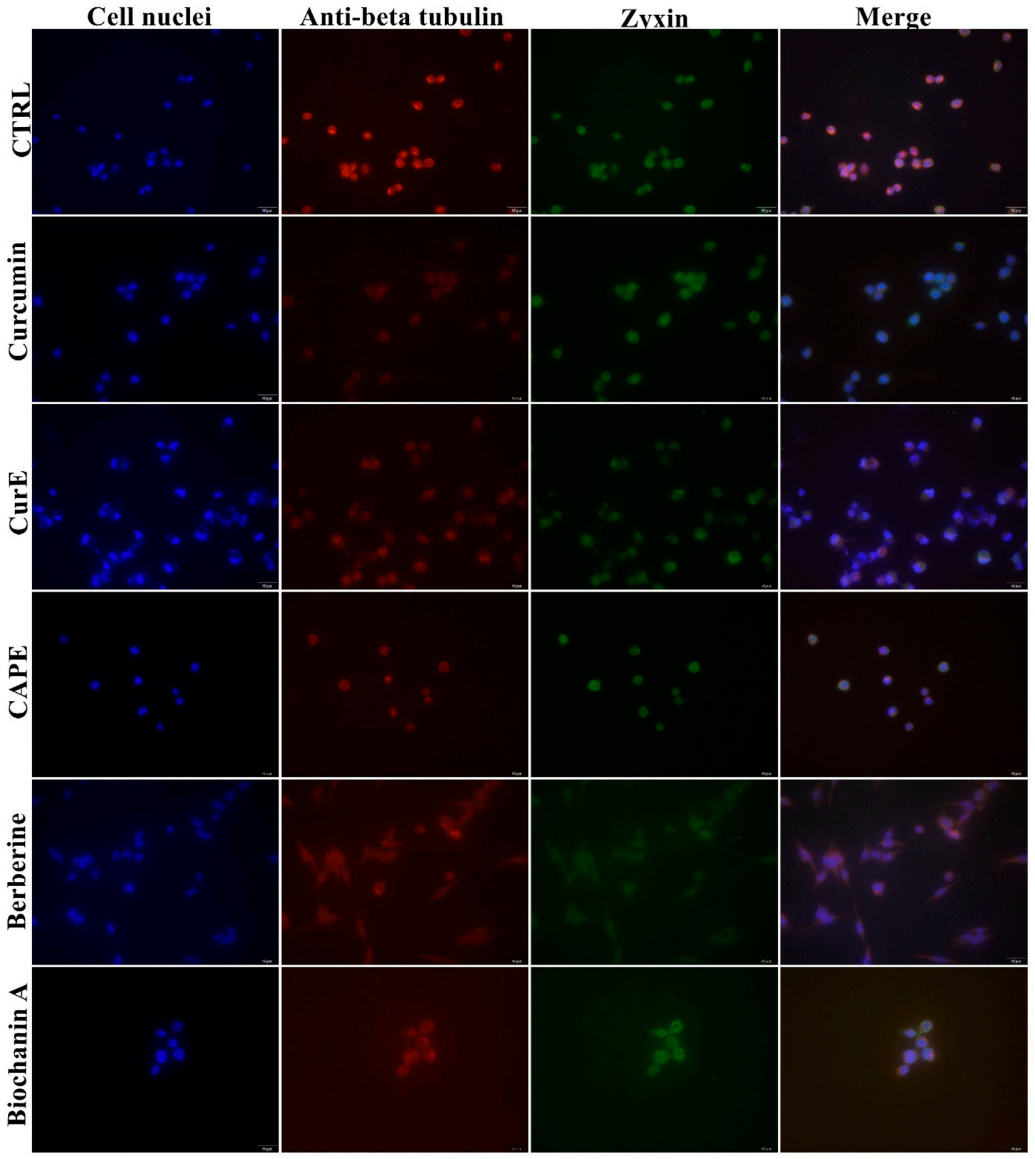
Presentation of the effect of curcumin (20 µM), CurE (2.5 µM), CAPE (25 µM), berberine (50 µM) and biochanin A (5 µM) on WEHI-164 cells after 24 h of incubation. Nuclei are stained blue with DAPI, zyxin is stained green with AlexaFluor488 and anti-beta tubulin antibody is stained red with AlexaFluor594. Scale bar = 20 μm.

The distribution of proteins responsible for cytoskeleton organization - zyxin and tubulin, was detected using the immunofluorescence method, and is shown in Figure 13 for normal cells, and Figure 14 for WEHI-164 cells. Control group showed normal distribution and intensity of the tubulin-related (anti-beta tubulin) and zyxin-related fluorescence, indicating standard cytoskeletal organization. Curcumin exposure resulted in a noticeable decrease in fluorescence intensity for both tubulin and zyxin, suggesting cytoskeletal disruption. When cells were treated by CurE, a reduction in intensity and some disorganization was noted, though less pronounced as compared to Curcumin. CAPE treatment caused significant reduction in fluorescence intensity, indicating major cytoskeletal disruption. In cells incubated with berberine a lower fluorescence intensity and potential structural changes suggesting cytoskeletal disturbance were observed. Biochanin A incubation induced a reduced intensity and altered distribution of cytoskeletal proteins, indicating reorganization. The obtained results indicate that the treatments with Curcumin, CurE, CAPE, Berberine, and Biochanin A induce disrupt cytoskeletal organization in cancer cells, potentially impairing cell division and motility.

**FIGURE 15:**
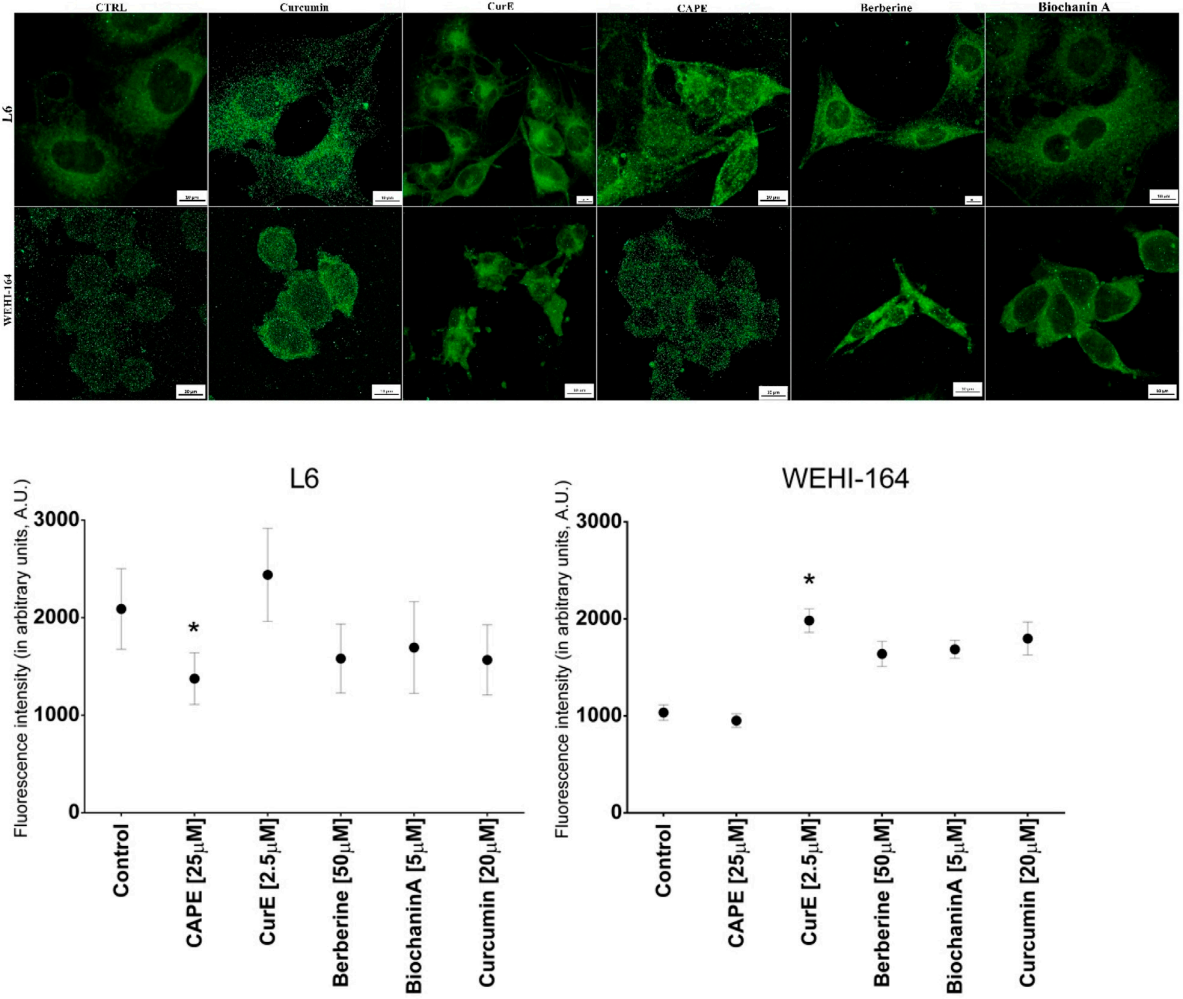
Presentation of the effect of curcumin (20 µM), CurE (2.5 µM), CAPE (25 µM), berberine (50 µM) and biochanin A (5 µM) on L6 and WEHI-164 cells after 24 h of incubation using CLSM **(A)**. Muscle FBPase stained green with AlexFluor488. Scale bar = 10 μM. Intensity of fluorescence of AlexaFluor488 (A11029) in L6 and WEHI-164 cells in arbitrary units **(B)**; *p < 0.05.

Immunofluorescent analysis of FBPase is demonstrated in Figure 15 A and B.

In the cell L6 line, only in samples incubated with CurE the fluorescence was more intense compared to the control. In the analysis performed on the WEHI-164 cell line, the fluorescence was significantly more intense compared to the control in samples after incubation with CurE, berberine, curcumin and biochanin A (Figure 15 B). Moreover, FBPase localization ceased to be granular, becoming more homogeneous, suggesting that exposure to CurE reduced FBPase interactions with the tubulin cytoskeleton and/or mitochondria. FBPase released from the protein complex is more easily accessible to antibodies. And therefore, the FBPase-related fluorescence is more intense, as it was observed in cancer cells, after CAPE and biochanin.

### 3.7 Glycogen levels regulation by natural compounds

**FIGURE 16:**
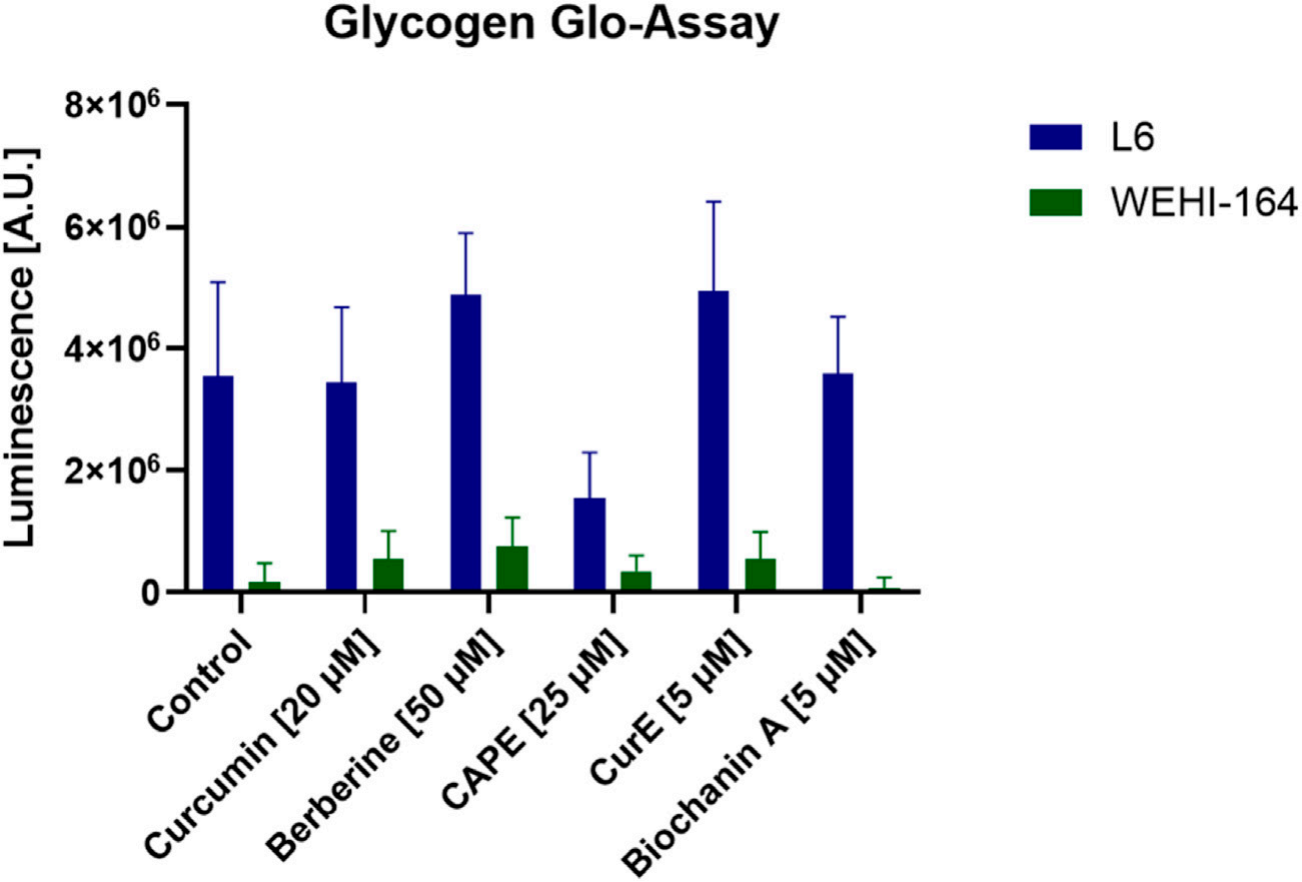
Measurement of glycogen levels in L6 and WEHI-164 cells in arbitrary units. Notes: (mean ± SD) N = 3.

The evaluation of glycogen levels in L6 (rat myoblast) and WEHI-164 (mouse fibrosarcoma) cells after exposure to natural anticancer drugs may help to understand the metabolic impact of these treatments. Specifically, it enables to investigate whether these drugs disrupt glycogen metabolism, which could lead to reduced glycolytic flux and subsequent energy deprivation in cancer cells. The results of our analysis are shown in Figure 16. These data indicate that L6 cells have higher baseline glycogen levels compared to WEHI-164 cells. This suggests a possible difference in glycogen metabolism or storage capacity between the 2 cell lines. In the case of the drug exposure, all tested compounds reduced glycogen levels in both L6 and WEHI-164 cells, with varying degrees of impact. Berberine and Biochanin A treatment resulted in the most significant reduction in glycogen levels in L6 cells, suggesting a strong inhibitory effect on glycogen storage or synthesis. Curcumin and CAPE moderately reduced glycogen levels in L6 cells. CurE had the least effect on glycogen levels in L6 cells, indicating it may be less effective at altering glycogen metabolism in this cell line. Interestingly, in WEHI-164 cells, all treatments resulted in minor reductions in glycogen levels, but overall the levels remained low compared to L6 cells.

The reduction in glycogen levels may indicate that these compounds inhibit glycogen synthesis or promote glycogen degradation, leading to decreased energy reserves in the cancer cells. This could contribute to the observed cytotoxic effects, as cancer cells are deprived of a crucial energy source, impairing their proliferation and survival.

### 3.8 Branched-chain amino acids levels regulated by natural compounds

**FIGURE 17:**
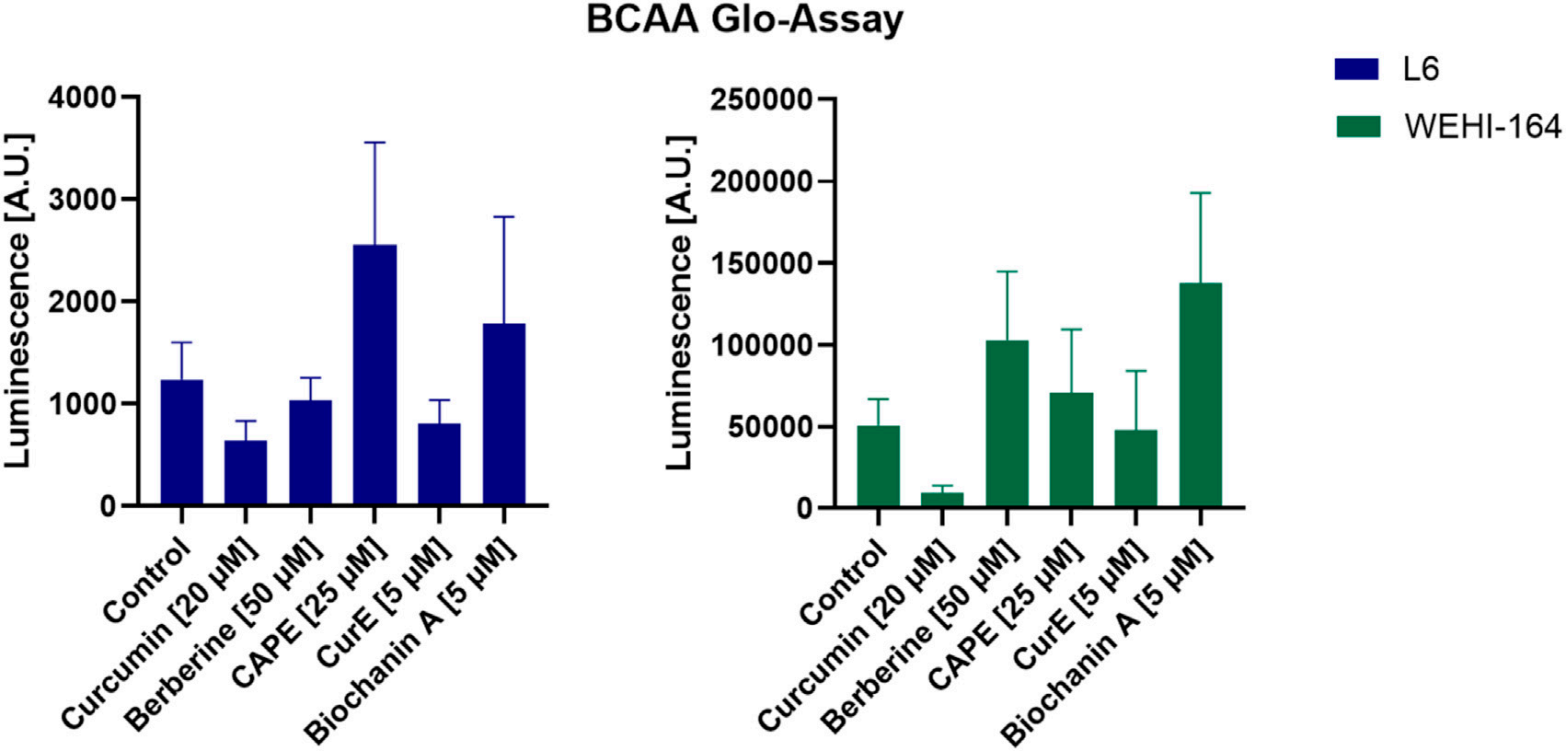
Measurement of BCAA levels in L6 and WEHI-164 cells in arbitrary units. Notes: (mean ± SD) N = 3.

Branched-chain amino acids (BCAA), including valine, leucine, and isoleucine, are pivotal amino acids for tumour formation in a range of human malignancies (Xu et al., 2023). A growing body of evidence from scientific studies indicates that there are significant alterations in the metabolism of BCAA and their associated proteins in a number of different cancer phenotypes. This suggests that disturbances in the metabolism of BCAA may become a significant factor in the reprogramming of cancer metabolism (Jung et al., 2021). Elevated levels of branched-chain amino acids (BCAA) have been linked to the suppression of tumour growth, suggesting their potential as anticancer agents (Martin et al., 2020). The findings are illustrated in Figure 17. BCAA levels were found to be significantly elevated in cancer cells in comparison to normal muscle cells. The highest concentration of BCAA was observed in WEHI-164 cells following incubation with biochanin A and berberine. The lowest level of BCAA was observed in the cancer cells following incubation with curcumin. In the case of L6 cells, the highest level of luminescence is observed following the use of biochanin A. The remaining compounds demonstrate a lower level of luminescent activity in comparison to biochanin A, which may suggest that this compound exerts the strongest effect on these cells.

### 3.9 Wound healing assay

**FIGURE 18:**
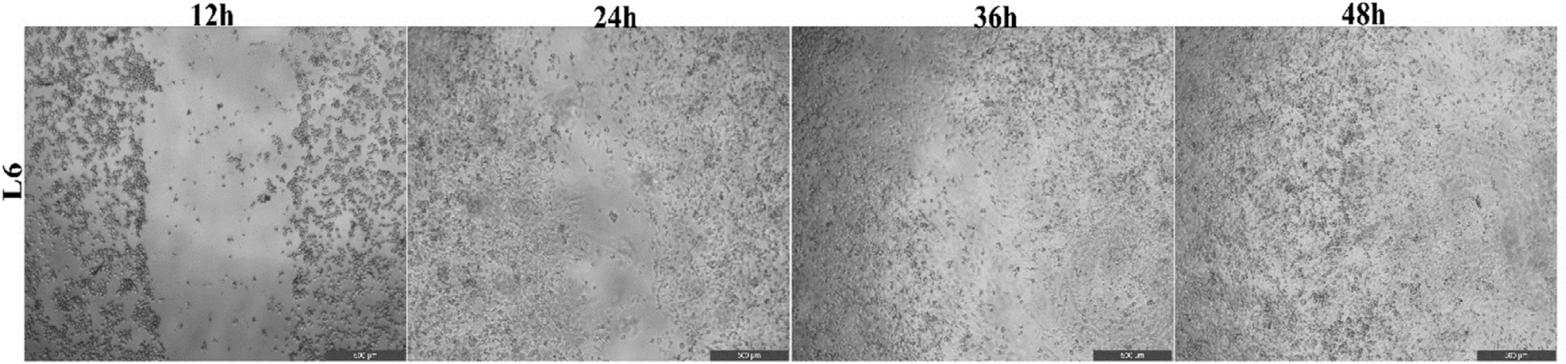
Closure of the wound gap in L6 cells after 12, 24, 36 and 48 h after incubation with CurE [2.5 µM].

**FIGURE 19:**
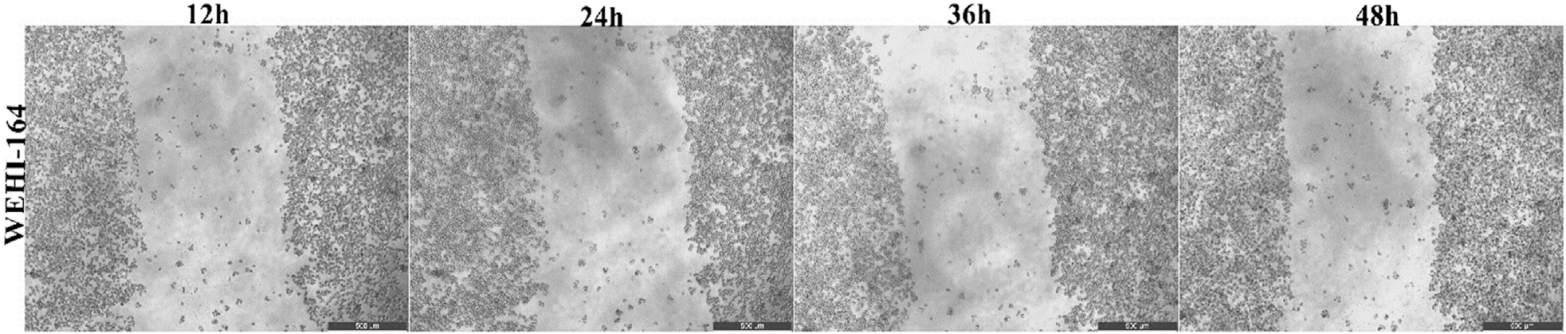
Closure of the wound gap in WEHI-164 cells after 12, 24, 36 and 48 h after incubation with CurE [2.5 µM].

The assay was performed to assess whether berberine (50 µM), CAPE (25 µM), CurE (2.5 µM), curcumin (20 µM) and biochanin A (5 µM) affected the migration of L6 and WEHI-164 cells. Progress in wound healing was analysed after 12, 24, 36 and 48 h with a microscope (magnification ×20). The following photographs show the process of 500 μm cell-free wound gap closure (Figures 18, 19).

Based on the obtained data (Tables 3, 4), it can be concluded that the analysed substances inhibited the closing of the wound gap in cancer cells from the WEHI-164 line. After 48 h of incubation with CurE, the gap was closed by 26.12% compared to the control, which was the lowest result of all the analysed ones. After 48 h of incubation of WEHI-164 cells with curcumin, the gap was closed by 37.12% and after incubation with CAPE by 39.12% compared to the control. The strongest wound closure was observed after 48 h of incubation with biochanin A, where the gap was closed by 72.24%. When the normal L6 cells was incubated substances, the gaps were closed to a very similar extent as in the control case. The results that differed the most from those obtained under control conditions were obtained after 48 h of incubation with berberine, where the gap was closed by 87.12%.

**TABLE 3:** Percentage of L6 cells wound gap closure after incubation of the cells with the analyzed substances.

Concentration	Incubation time			
	12 h	24 h	36 h	48 h
Control	24.32%	47.22%	68.82%	99.24%
Berberine [50 µM]	8.22%	23.34%	51.09%	87.12%
Caffeic acid phenethyl ester (CAPE) [25 µM]	22.98%	41.12%	72.02%	91.14%
Cucurbitacin E (CurE) [2.5 µM]	23.92%	49.12%	87.24%	93.12%
Curcun1in [20 µM]	12.93%	42.12%	74.93%	92.22%
Biochanin A [5 µM]	22.21%	34.12%	64.33%	91.12%

**TABLE 4:** Percentage of WEHI-164 cells wound gap closure after incubation of the cells with the analysed substances.

Concentration	Incubation time			
	12 h	24 h	36 h	48 h
Control	20.91%	49.82%	74.23%	99.51%
Berberine [50 µM]	14.31%	36.09%	44.47%	57.72%
Caffeic acid phenethvl ester (CAPE) [25 µM]	10.24%	16.98%	26.12%	39.12%
Cucurbitacin E (CurE) [2.5 µM]	9.24%	17.22%	21.02%	26.12%
Curcumin [20 µM]	7.98%	17.76%	24.98%	37.12%
BiochaninA [5 µM]	14.45%	34.76%	57.12%	72.24%

## 4 Discussion

The present study investigated the effects of several natural NF-κB inhibitors (berberine, curcumin, biochanin A, CurE and CAPE) on fibrosarcoma (WEHI-164) and normal muscle cells (L6). The results showed that these compounds had a stronger cytotoxic effect on cancer cells compared to normal cells, suggesting their potential as effective anti-cancer agents. Moreover, such a selective toxicity is essential to minimize side effects in cancer therapy. Apoptosis was induced in WEHI-164 cells by all compounds, with CAPE and curcumin showing significant apoptotic effects after 48 h of incubation. Other studies revealed that curcumin and berberine can inhibit cancer cell migration and invasion by modulating cytoskeletal dynamics and cellular adhesion properties (Pavan et al., 2016). Our results of viability assays confirmed the selective cytotoxicity of these compounds, with cancer cells being more adversely affected than normal muscle cells. Molecular docking revealed that CAPE, biochanin A and CurE inhibited actin polymerization, indicating their potential role in disrupting the cytoskeleton of cancer cells. Berberine’s interaction with intracellular kinases, such as AMPK, suggests a unique mechanism where it modulates cellular energy balance and metabolic pathways (Kim et al., 2009). It has been shown that curcumin is able to inhibit actin polymerization and reduce the metastatic potential of cancer cells (Sadeghi et al., 2023). Similarly, CAPE has been shown to interfere with microtubule dynamics, leading to cell cycle arrest and apoptosis in cancer cells (Chuu et al., 2012). Immunocytochemical analysis revealed increased expression of caspase-3 and PARP-1 in WEHI-164 cells after treatment with the tested compounds, supporting the assumption of induction of apoptosis. Transmission electron microscopy revealed cellular stress and vacuolization in cancer cells treated with the compounds, with more pronounced effects observed in cancer cells compared to normal cells.

We also verified glycogen and branched-chain amino acids (BCAA) levels. We found that all tested compounds reduced glycogen levels. In cancer cells, glycogen metabolism can support rapid proliferation by providing a quick energy source and intermediates for biosynthetic pathways. It can also impact the Krebs Cycle. The Krebs cycle (TCA cycle) relies on acetyl-CoA derived from glycolysis and other sources. Reducing glycogen levels could reduce glycolytic flux and thereby inhibit the TCA cycle, potentially starving cancer cells of energy and biosynthetic precursors (Halabe Bucay, 2007). Cancer cells often rely on aerobic glycolysis (Warburg effect) rather than oxidative phosphorylation (Pelicano et al., 2006). However, inhibiting glycogen could still stress their metabolism, especially under fluctuating glucose conditions. Compounds that decrease glycogen levels in cancer cells can potentially inhibit their growth by reducing available glucose for glycolysis and the TCA cycle. A decrease in glycogen levels can lead to reduced ATP production, slower proliferation, and increased sensitivity to other metabolic stresses or treatments. Our findings highlight the potential of natural compounds to disrupt cancer cell metabolism and cytoskeletal organization, contributing to their antiproliferative and cytotoxic effects. For anticancer therapy, targeting glycogen metabolism to decrease glycogen levels in cancer cells could be beneficial (Khan et al., 2020). This approach could (i) starve cancer cells, i.e., reduce energy supply and biosynthetic precursors from glycolysis and the TCA cycle (ii) increase stress and make cancer cells more susceptible to metabolic inhibitors or other treatments. Natural compounds which are potential NF-κB inhibitors can selectively induce apoptosis, disrupt cytoskeletal organization, and impair glycogen metabolism in fibrosarcoma cells while sparing normal muscle cells. These compounds’ ability to reduce glycogen levels, inhibit cell migration, and interact with cytoskeletal proteins underscores their potential as multifaceted anti-cancer agents. By targeting both metabolic and structural components of cancer cells, these natural compounds offer a promising approach for cancer therapy, enhancing the effectiveness of existing treatments and reducing side effects.

Berberine has been combined with cisplatin (CPP) (Xiong et al., 2022) or doxorubicin (DOX) (Almatroodi et al., 2022) in studies showing that it enhances the cytotoxicity of cisplatin in cancer cells while protecting normal cells from damage by reducing oxidative stress. This combination has been particularly investigated in lung and ovarian cancers. Similarly CAPE also enhanced anticancer efficacy of DOX (Liang et al., 2023). CAPER revealed promising activity in resistant breast or colon cancers. Other studies showed that curcumin can be used with paclitaxel by inhibiting NF-κB and reducing cancer cell resistance, particularly in breast and ovarian cancer (Aggarwal et al., 2005). Cur was also combined with 5-Fluorouracil (5-FU) demonstrating synergistic effects in colon cancer (Xu et al., 2020). Also CuE was combined with DOX (Si et al., 2019) impaired AKt activation in gastric cancer, or with CPP inhibited the growth of human breast cancer cells *in vitro* (Lan et al., 2013). Thus, these combinations underscore the potential of using natural compounds alongside classical chemotherapeutic agents to improve treatment outcomes, reduce drug resistance, and minimize side effects. Thus, our results contribute to the growing knowledge in the use of natural compounds for cancer treatment. However, future studies should further elucidate the molecular mechanisms involved and explore the synergistic effects of combining these natural inhibitors with conventional chemotherapy and radiotherapy.

## 5 Conclusion

Results of the study support the hypothesis that natural NF-κB inhibitors can selectively target cancer cells, reducing their viability and inducing apoptosis, while sparing normal cells. This selectivity is critical for the development of safer cancer treatments. The findings provide a rationale for the potential use of these natural compounds in anti-cancer therapy, particularly for fibrosarcoma. Their ability to inhibit key metabolic pathways and induce apoptosis in cancer cells without significantly affecting normal cells positions them as promising candidates for further preclinical and clinical evaluation. Although the *in vitro* results are encouraging, they may not fully reflect the intricate *in vivo* environment. The study is constrained by its exclusive focus on cell lines, consequently, further research involving animal models and clinical trials is required to validate these findings. Further research is required to elucidate the detailed mechanisms of action of these compounds *in vivo*, their pharmacokinetics, and potential synergistic effects with existing chemotherapeutic agents. The findings of this study demonstrate the efficacy of natural NF-κB inhibitors in targeting fibrosarcoma cells, offering a promising direction for the development of more effective and less toxic cancer therapies.

## Data Availability

The raw data supporting the conclusions of this article will be made available by the authors, without undue reservation.
